# Lithium-Ion Dynamic Interface Engineering of Nano-Charged Composite Polymer Electrolytes for Solid-State Lithium-Metal Batteries

**DOI:** 10.1007/s40820-025-01899-7

**Published:** 2025-08-29

**Authors:** Shanshan Lv, Jingwen Wang, Yuanming Zhai, Yu Chen, Jiarui Yang, Zhiwei Zhu, Rui Peng, Xuewei Fu, Wei Yang, Yu Wang

**Affiliations:** 1 College of Polymer Science and Engineering, National Key Laboratory of Advanced Polymer Materials, Sichuan University, Chengdu, 610065 People’s Republic of China; 2 Analytical and Testing Center, Sichuan University, Chengdu, 610065 People’s Republic of China

**Keywords:** Charged nanofillers, Nanocomposite polymer electrolyte, Dynamic lithium ion interface, Solid ion-conductors, Solid-state lithium-metal battery

## Abstract

Composite polymer electrolytes (CPEs) offer a promising solution for all-solid-state lithium-metal batteries (ASSLMBs). However, conventional nanofillers with Lewis-acid–base surfaces make limited contribution to improving the overall performance of CPEs due to their difficulty in achieving robust electrochemical and mechanical interfaces simultaneously. Here, by regulating the surface charge characteristics of halloysite nanotube (HNT), we propose a concept of lithium-ion dynamic interface (Li^+^-DI) engineering in nano-charged CPE (NCCPE). Results show that the surface charge characteristics of HNTs fundamentally change the Li^+^-DI, and thereof the mechanical and ion-conduction behaviors of the NCCPEs. Particularly, the HNTs with positively charged surface (HNTs^+^) lead to a higher Li^+^ transference number (0.86) than that of HNTs^−^ (0.73), but a lower toughness (102.13 MJ m^−3^ for HNTs^+^ and 159.69 MJ m^−3^ for HNTs^−^). Meanwhile, a strong interface compatibilization effect by Li^+^ is observed for especially the HNTs^+^-involved Li^+^-DI, which improves the toughness by 2000% compared with the control. Moreover, HNTs^+^ are more effective to weaken the Li^+^-solvation strength and facilitate the formation of LiF-rich solid–electrolyte interphase of Li metal compared to HNTs^−^. The resultant Li|NCCPE|LiFePO_4_ cell delivers a capacity of 144.9 mAh g^−1^ after 400 cycles at 0.5 C and a capacity retention of 78.6%. This study provides deep insights into understanding the roles of surface charges of nanofillers in regulating the mechanical and electrochemical interfaces in ASSLMBs.

**Supplementary Information:**

The online version contains supplementary material available at 10.1007/s40820-025-01899-7.

## Highlights

- The surface charge characteristics of halloysite nanotubes (HNTs) are manipulated to engineer the Li^+^-dynamic interface (Li^+^-DI) in composite polymer electrolytes.
- Surface charge characteristics of HNTs generate pronounced impact on not only the ionic/mechanical properties of the composite electrolytes, but also the formation and composition of solid–electrolyte interphase (SEI) layer.
- HNTs^+^-supported Li^+^-DI exhibits an anion-rich Li^+^-solvation structure and soft-and-tough mechanical interface, leading to LiF-rich SEI layer and improvement of toughness by over 2000% compared with the control.

**Supplementary Information:**

The online version contains supplementary material available at 10.1007/s40820-025-01899-7.

## Graphical abstract

**Figure:**
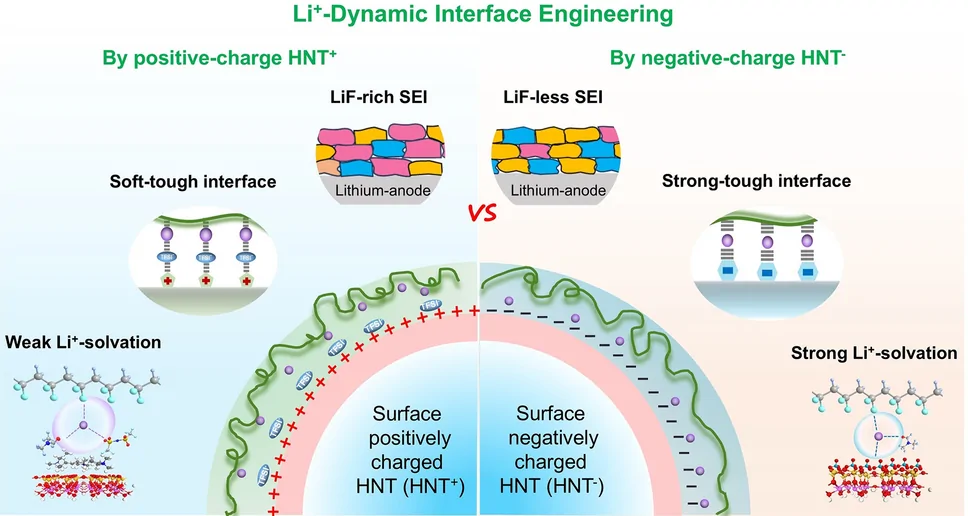

## Introduction

Solid-state lithium-metal batteries (SSLMBs) are promising to become the energy storage systems in the future. However, their practical applications are severely hindered by the unstable electrode/electrolyte interface during operation, as represented by uncontrolled lithium dendrite growth, continuous solid–electrolyte interphase (SEI) breakage/formation, and mechanical failure of the electrolyte membrane eventually [1–4]. Compared to highly-conductive but fragile ceramic electrolytes, polymer electrolytes are advantageous in processability, flexibility, and interface contact. To overcome the abovementioned electrochemical and mechanical interface issues, polymer electrolyte membranes should not only have high ionic conductivity and mechanical properties, but also capability to electrochemically stabilize the interface with electrode particularly with the lithium-metal anode. However, these essential properties face critical trade-offs because the ion-conduction, mechanical properties, and interface stability do not share the same compositions or structures.

It is well known that ionic conductivity is the foremost property for polymer electrolytes but remains below 10^–5^ S cm^−1^ at room temperature, restricting them from practical applications. The ion-conduction ability of polymer electrolytes strongly relies on the segment motion, which is contradictory to increasing their mechanical properties. As such, to increase the ionic conductivity while maintaining mechanical properties, polymer blending [5–7], crosslinking [8–11], and nanofiller compositing (e.g., metal–organic frameworks and halloysite nanotubes) [12–16] are the effective modification strategies. Among them, compositing nanofillers with polymer electrolytes can potentially increase the ionic conductivity via creating more ion-conduction pathways at the polymer–nanofiller interface, while reinforcing the mechanical properties [17, 18]. In recent years, surface functionalization and alignment of nanofillers [19], as well as construction of 3D continuous filler skeletons [20] have demonstrated to substantially improve the ionic conductivity close to 10^–3^ S cm^−1^ at room temperature. However, due to the poor polymer–nanofiller interface compatibility, the mechanical strength of modified composite polymer electrolytes often remains below 5 MPa [21]. Despite that grafting polymer onto the nanofiller [22], silane coupling agent [23], etc., are effective in improving the interface compatibility, the increase of both mechanical strength and ionic conductivity is limited to < 10 MPa and < 1 × 10^–4^ S cm^−1^, respectively, due to the blocked ion transport at the nanofiller–polymer interface. Therefore, searching for new nanofiller modification strategies to simultaneously enhance the ion-transport kinetics and compatibility of the nanofiller–polymer interface is urgently needed.

To improve the electrochemical stability of polymer electrolytes with lithium-metal anode, besides the design of chain structure [24], tremendous efforts focus on regulating the solid–electrolyte interphase (SEI) composition and structure by applying various additives due to their cost-effectiveness and universality. Namely, fluorine-containing solvents [25–27], dual-salt additive [28, 29], and high dielectric constant solvents [30] have been added into the polymer electrolytes to increase the electrochemical stability. These additives interact with lithium metal prior to the polymer electrolyte, generating stronger SEI layers such as the ones with LiF-rich component [31–34]. This effectively stabilizes the interface between the electrolyte and lithium metal by either enhancing the mechanical strength of the SEI layer or accelerating the ion transfer across the SEI layer. Nonetheless, the addition of the small molecule additives deteriorates mechanical properties of the polymer matrix due to the plasticizing effect, which undermines the stabilizing efficacy for the electrolyte/electrode interface in the long run.

To conquer the above challenges, we regulate and strengthen the surface charges of halloysite nanotubes (HNTs) to not only enhance the ion-transport kinetics and compatibility of the HNT/polymer interface but also assist the formation of a stronger SEI layer for the lithium-metal anode. It is found that the type and strength of the surface charge play critical roles in regulating the Li^+^-dynamic interface (Li^+^-DI) in the composite electrolyte formed at the polymer/HNT interface. It is noted that under electric field, Li^+^ ions are movable at the polymer/HNT interface, which dynamically bridges polymer chains and HNTs, and thus, we define it as the Li^+^-DI. The Li^+^-DI fundamentally controls the ion-transport behaviors, mechanical properties, and even the SEI formation process. Meanwhile, the 1D nanotube structure provides strong adsorption and confinement effect of residual solvents, helping to form fast ion-conduction pathway inside the nanotube. The charged HNTs exhibit better efficacy to increase the ionic conductivity, mechanical properties, and electrochemical stability of the resultant composite polymer electrolytes, in comparison with the pristine HNTs. The regulation, characterization, and understanding of the surface charge-enhanced polymer/HNT and electrolyte/electrode interfaces are displayed below.

## Experimental Section

### Materials

Polyvinylidene fluoride (PVDF, MTI Corp.), poly(diallyldimethylammonium chloride) (PDDA, Shanghai Aladdin Biochemical Technology Co., Ltd.), lithium bis(trifluoromethanesulfonyl)imide (LiTFSI, Shanghai Aladdin Biochemical Technology Co., Ltd), halloysite nanotubes (HNTs, Shanghai Aladdin Biochemical Technology Co., Ltd.), LiFePO_4_ (LFP, Guangdong Canrd New Energy Technology Co., Ltd.), LiNi_0.8_Co_0.1_Mn_0.1_O_2_ (NCM811, Guangdong Canrd New Energy Technology Co., Ltd.), and carbon black (Super C65, Guangdong Canrd New Energy Technology Co., Ltd.) were all used as received.

### Preparation of Negatively Charged HNTs (HNTs.^−^)

The HNTs^−^ were prepared by sodium hexametaphosphate modification as previously reported [35]. In brief, pristine HNTs were first dispersed in 20 wt.% ethanol solution with a solid content of 10 wt.%. After standing for 24 h, the dispersion was washed by water and centrifugated at 7000 r min^−1^ for 3 min. The obtained sediment was dried in a vacuum oven for 12 h at 80 °C and grinded into powders. The resulting HNTs were then dispersed in 20 wt.% ethanol solution with 1 wt.‰ sodium hexametaphosphate under stirring for 1 h [36]. After standing for 24 h, the supernatant was washed by water for multiple times by centrifugation at 12,000 r min^−1^ for 5 min to remove excessive surfactant. Then, the sediment was dried in a vacuum oven for 12 h at 80 °C and grinded to powder to obtain HNTs^−^.

### Preparation of Positively Charged HNTs (HNTs.^+^)

The HNTs^**+**^ were prepared by poly(diallyldimethylammonium chloride) modification as follows. HNTs were first dispersed in an ethanol solution (ethanol/water: 20:80, wt/wt) with a solid content of 10 wt.%. After stirring for 24 h, the dispersion was washed by water and centrifugated at 7000 r min^−1^ for 5 min. The obtained sediment was dried in a vacuum oven for 24 h at 80 °C and grinded into powders. The resulting HNTs were then dispersed in water with 10 wt.% PDDA under stirring for 6 h. PDDA was washed by water multiple times by centrifugation at 20,000 r min^−1^ for 5 min to remove residual PDDA. Then, the precipitates were dried in a vacuum oven for 24 h at 80 ℃ and grinded to powder to obtain HNTs^+^.

### Preparation of Composite Polymer Electrolyte Membranes

Before use, PVDF powders were dried in a vacuum oven at 60 °C for 12 h to remove moisture. The PVDF powder was dissolved in N-dimethylformamide (DMF) solvent with a solid content of 10 wt.% to form a homogenous solution. Then, LiTFSI was added to the solution according to a PVDF:LiTFSI weight ratio of 3:1. Various HNTs were dispersed in DMF to obtain the dispersion. Then, certain amount of HNT dispersions were added to the PVDF-LiTFSI solution and stirred for 12 h. The mixture was scraped on a glass substrate with controlled thickness of about 40 μm and pre-dried at 80 °C to form a solid membrane. The membrane was further dried in a vacuum oven for 24 h at 60 °C to remove residual solvent and moisture to obtain the electrolyte membrane.

### Material Characterizations

#### Morphology and Molecular Interactions

The morphology of the free surface and cross-section of the samples was characterized by using polarized light microscopy (OLYMPUS-BX51, Japan), scanning electron microscopy (SEM, Hitachi Regulus8220, Japan), and transmission electron microscope (TEM, Thermo Scientific Talos FEI 200, USA). Raman spectra were obtained by using a Raman spectrometer (inVia Reflex, Renishaw, UK). The surface elements of cycled lithium anodes were probed by using X-ray photoelectron spectroscopy (Thermo, Scientific K-Alpha, USA). The X-ray Diffraction (XRD) patterns were obtained by using Rigaku X-ray diffractometer Ultima Ⅳ with Cu-Kα radiation (λ = 1.5418 Å). The thermal stability was measured by using thermogravimetric analysis measurement (TGA, Mettler Toledo TGA 2 system, Mettler Toledo Corp., Switzerland) from 30 to 800 °C at a 10 °C min^−1^ ramping rate under an N_2_ atmosphere. The solid-state ^7^Li magic angle spinning (MAS) nuclear magnetic resonance spectroscopy was recorded on NMR spectrometer (Agilent 600 MHz, USA). The solid-state ^19^F magic angle spinning (MAS) nuclear magnetic resonance spectroscopy was recorded on NMR spectrometer (Agilent 400 MHz, USA). Chemical shifts were given in ppm relative to tetramethyl silane. The crystallization behavior was investigated by using a differential scanning calorimetry (DSC, TA Instruments Q20, Milford, MA, USA) from 30 to 200 °C at a heating rate of 10 °C min^−1^ in an N_2_ atmosphere. The degree of crystallinities (χ_c_) was calculated using the below equation:

1 $${x}_{c}=\frac{{\Delta H}_{m}}{{\Delta H}_{m}^{0} \cdot f}\times 100\%$$

where ${\Delta H}_{m}$ was the melting enthalpy of the sample; ${\Delta H}_{m}^{0}$ was the enthalpy of fusion of complete crystalline PVDF with a value of 102.7 J g^−1^, and f was the weight fraction of PVDF in the composites [1].

#### Surface Charge and Mechanical Properties

The surface charge of HNTs was examined by using Malvern Zetasizer (Nano ZS UK). The tensile properties of the membranes were measured by universal material testing machine (Ultima Ⅳ, Japan) with a constant tensile rate of 50 mm min^−1^. All the simple size (5 mm × 30 mm, length × width), bending length, and movement speed were fixed.

#### Electrochemical Measurements

The ionic conductivities were measured by electrochemical impedance spectroscopy (EIS) through an electrochemical workstation (Princeton-ParStat400, USA). The frequency ranged from 1 MHz to 0.1 Hz with an amplitude voltage of 10 mV from 25 to 80 °C. The polymer electrolyte membranes were sandwiched between two stainless steel (SS, 16 mm in diameter) blocking electrodes with an SS|polymer electrolyte|SS configuration. The ionic conductivity (σ) was calculated according to the below equation:

2 $$\sigma =\frac{L}{RS}$$

where *R* and *L* referred to the bulk resistance and the thickness of electrolytes, respectively, and *S* referred to the area of SS.

The electrochemical stability of the electrolytes was tested via linear sweep voltammetry (LSV) from 0 to 6 V at a scanning rate of 1 mV s^−1^ in the Li|electrolyte|SS cells. The lithium-ion transference number (${t}_{{\mathrm{Li}}^{+}}$) was characterized by combining AC impedance and DC polarization techniques with a polarization voltage of 10 mV on Li||Li symmetrical cells at room temperature. The EIS before and after polarization was acquired in the frequency range of 1 MHz–1 Hz. The transference number was calculated according to the following equation:

3 $${t}_{{Li}^{+}}=\frac{{I}_{s}\left(\Delta V-{R}_{0}{I}_{0}\right)}{{I}_{0}\left(\Delta V-{R}_{\mathrm{s}}{I}_{\mathrm{s}}\right)}$$

where ${I}_{\mathrm{s}}$ and ${I}_{O}$ were the steady-state and initial current, respectively; ${R}_{0}$ and ${R}_{\mathrm{s}}$ referred to the resistance of the cell before and after polarization, respectively. All the impedance data were fitted and analyzed using the electrochemical impedance software Z-view (Scribner Associates Inc.). The dependence of ionic conductivities on temperature is fitted by the Arrhenius plots, and the activation energy can be calculated by the Arrhenius equation below:

4 $$\sigma =\mathrm{Aexp}\left(\frac{{-E}_{\mathrm{a}}}{RT}\right)$$

where A is the pre-exponential factor; ${E}_{\mathrm{a}}$ is the activation energy; R is the ideal gas constant (R = 8.314 J mol^−1^ K^−1^), and T is the temperature.

#### Electrode Preparation and Coin Cell Assembly

Preparation of the cathode: The LiFePO_4_ (LFP) cathode was prepared by mixing LFP, carbon black, and PVDF binder solution in a weight ratio of 80:10:10. The LiNi_0.8_Co_0.1_Mn_0.1_O_2_ (NCM811) cathode was prepared by mixing NCM811, carbon black, and PVDF binder solution in NMP a weight ratio of 80:10:10, followed by casting the slurry on an Al foil and drying at 120 °C for 12 h. The cathode was cut into circular disks (12 mm in diameter). The active material loading was 3.5 mg cm^−2^ for the LFP cathode and 4 mg cm^−2^ for the NCM811 cathode. The thickness of the polymer electrolyte membranes was kept at 40–50 μm. The cells were assembled by using 2032-type coin cells in an argon-filled glovebox. Li||Li symmetric cells were assembled by sandwiching the electrolyte membranes between two Li chips.

All the cells were tested by Neware Battery measurement system with a voltage range of 2.5–3.65 V (LFP) or 2.5–4.2/2.8–4.4 V (NCM811) at room temperature. For cyclic voltammetry (CV) test, the voltage range of Li||Cu batteries was 0–2.5 V with a scan rate of 0.5 mV S^−1^. The EIS measurement was conducted by using the electrochemical workstation (ParStat400, Princeton, USA) in the frequency range of 1 MHz–1 Hz with an amplitude voltage of 10 mV at the open-circuit voltage. The average Coulombic efficiency of the Li||Cu cells over n cycles was calculated according to the below equation:

5 $$\mathrm{CE}=\frac{(n\times {Q}_{C}+{Q}_{F})}{(n\times {Q}_{C}+{Q}_{R)}}\times 100\%$$

Here ${Q}_{R}$ was used to deposit Li onto the Cu substrate first as a Li reservoir. ${Q}_{c}$ was used to cycle Li between working and counter electrodes for *n* cycles. After *n* cycles, a final exhaustive strip of the remaining Li reservoir was performed to the cut-off voltage. ${Q}_{F}$ referred to the quantity of Li remaining after cycling.

### Simulation Methods

#### Density Functional Theory Calculations

The Dmol3 module with the GGA/PBE functional, custom Grimme DFT-D parameters, and DNP 4.4 basis set in Material Studio software was used for density functional theory (DFT) calculations. The ESP charges were applied to optimize all molecules. The k-points were set at Gamma (1 × 1 × 1), and the convergence tolerance was set at 1.0 × 10^–5^ Ha, 2.0 × 10^–3^ Ha Å^−1^, and 5.0 × 10^–3^ Å for energy, maximum force, and maximum displacement, respectively.

The solvent energy was calculated by the following formula:

6 $${E}_{\mathrm{ads}}= {E}_{\mathrm{total}}-{E}_{1}- {E}_{2}$$

where *E*_total_, *E*_n_, and *E*_ads_ were the energy of total structure, the single molecule, and the adsorption energy, respectively [2].

#### TS-DFT

The Li^+^ diffusion path through different surfaces was calculated using the TS-DFT method with GGA/PBE functional, DFT-D2 correction, and DNP 3.5 basis set in the Dmol3 module. Migration energy was defined as the relative energy between adjacent high-symmetric Li^+^ adsorption positions along the migration path.

## Results and Discussion

### Concept of Li^+^-Dynamic Interface Engineering by Controlling Surface Charges

**Fig. 1:**
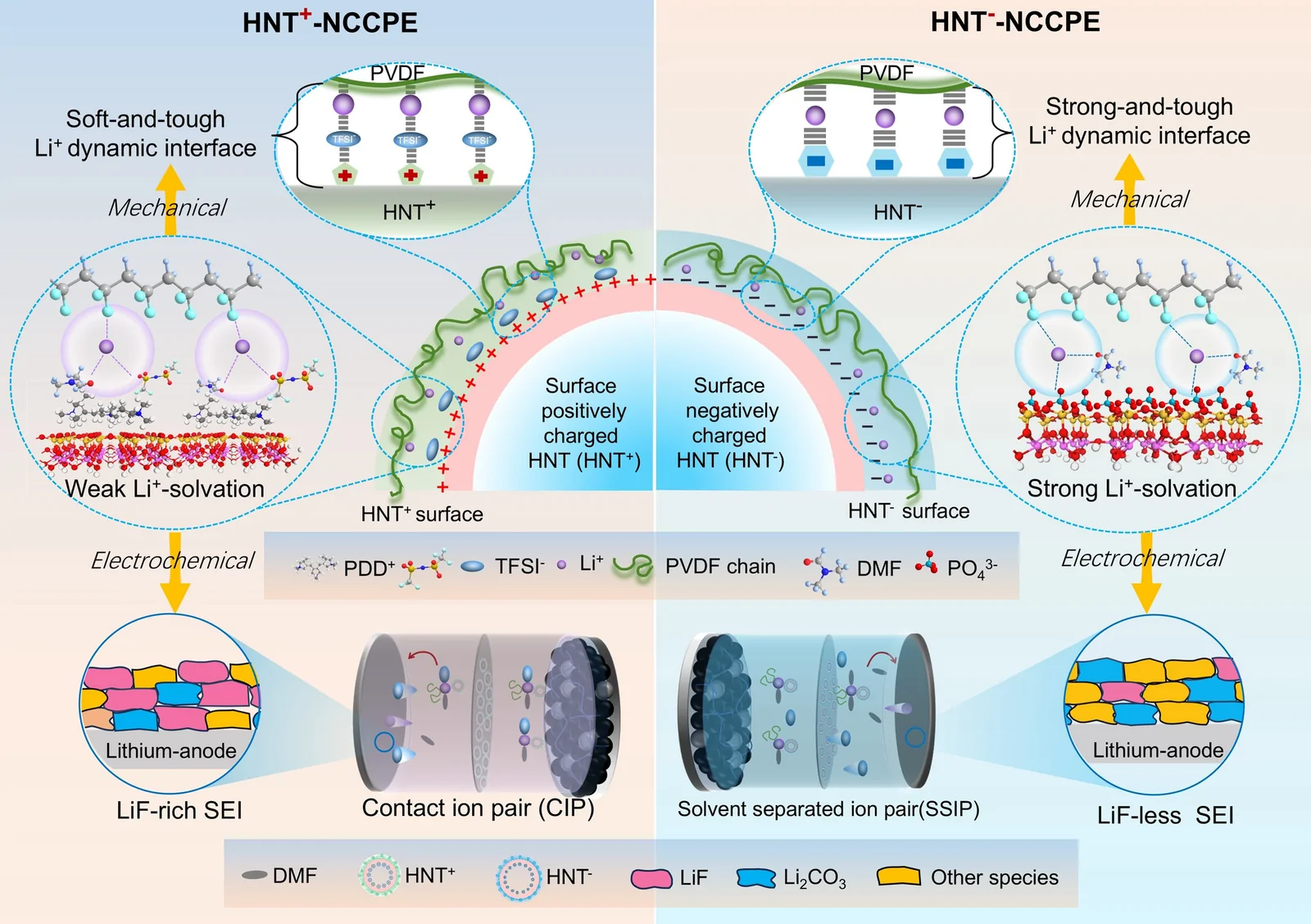
The concept of lithium-ion dynamic interface (Li^+^-DI) engineering in nano-charged composite polymer electrolytes (NCCPEs). The mechanical and electrochemical interfaces of NCCPEs are rationally regulated by the surface charge characteristics of halloysite nanotubes (HNTs) for all-solid-state lithium-metal batteries (ASSLMBs) (for details, see the text)

The polymer–nanofiller interface plays the pivotal role in the overall properties of composite electrolytes. Our previous study [35] revealed that increasing the strength of negative charge on HNT surface effectively enhances the interfacial interactions of HNT with ions and polyvinylidene fluoride (PVDF) chains, thus forming robust Li^+^-transport-friendly interface inside the composite polymer electrolytes. However, the detailed effects and mechanisms of surface charge characteristics of nanofillers on the ion-transport behaviors and mechanical properties of composite electrolytes have not been fully understood. In the current study, we prepared two types of charged HNTs with negative or positive surface charges, denoted as HNTs^−^ or HNTs^+^, respectively, to deeply uncover how the surface charge characteristics affect the ionic and mechanical behaviors with a focus on the polymer–nanofiller interface. As illustrated in Fig. 1, our results show that the resultant composite electrolytes with two types of surface charge-enhanced HNTs, namely, nano-charged composite polymer electrolytes (NCCPEs), exhibit quite different interface behaviors, i.e., Li^+^-DI behaviors, which fundamentally control the ion transport and mechanical properties of the NCCPEs. The charged HNTs, compared to the unmodified HNTs, effectively increase the electrostatic adsorption for Li^+^ or anion, helping enhance the interface bonding strength. A deeper investigation reveals that the interface strength of HNT^−^-NCCPE is higher than that of the HNT^+^-NCCPE. The Li^+^-solvation structure and thus the ion-transport and interface chemistry are also greatly changed by the surface charge characteristics of HNTs. An anion-involved weak Li^+^-solvation structure is formed at PVDF-HNT^+^ interface, which causes slower ion-transport kinetics but a more robust inorganic-rich SEI layer. On the contrary, the NCCPE-HNT^−^ obtains a strong Li^+^-solvation structure dominated by residual solvent (DMF) and PVDF chains, leading to faster ion-transport kinetics but weaker SEI layer.

**Fig. 2:**
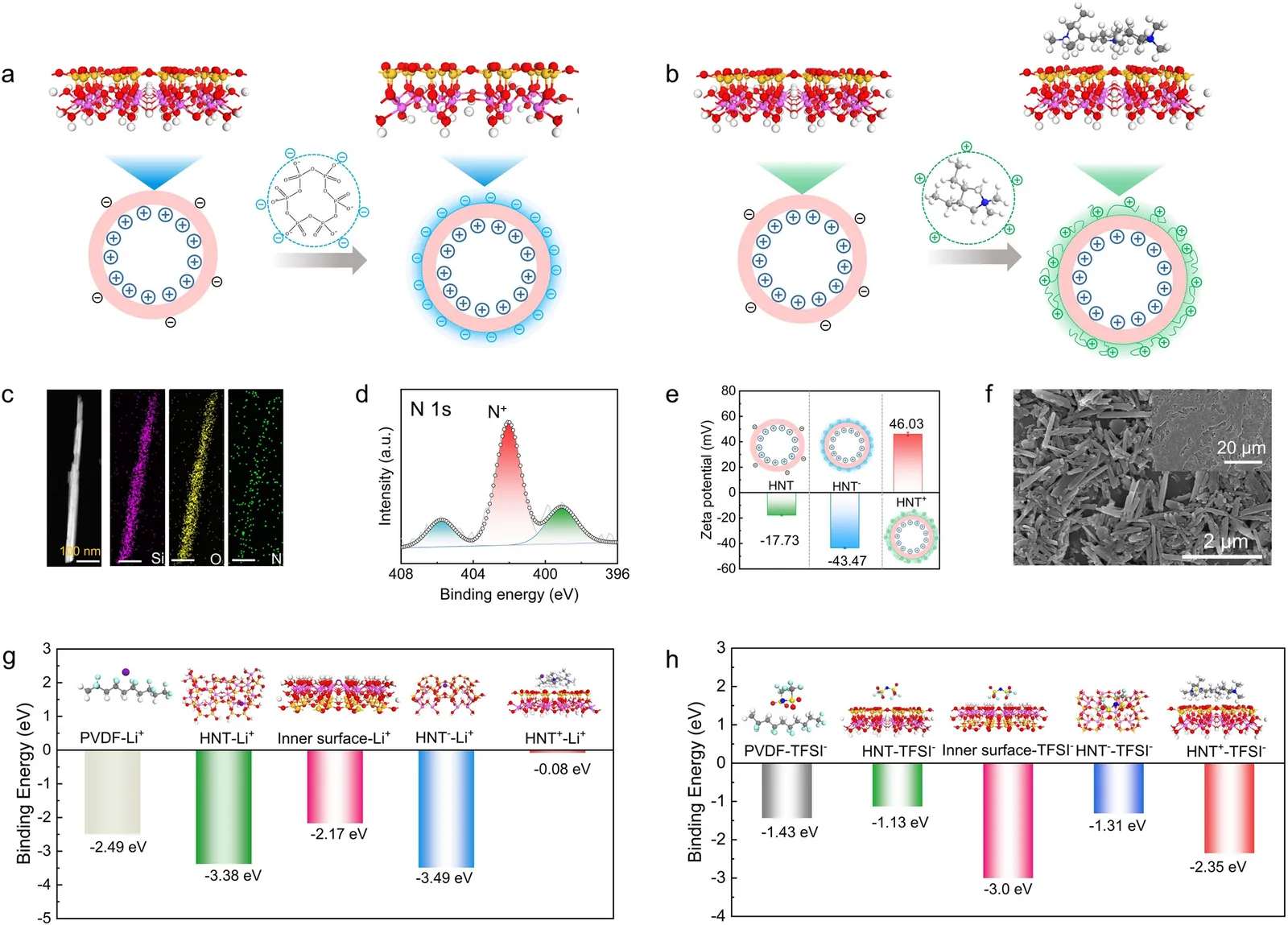
Surface charge regulation for controlling the interactions between charged HNTs and the ions of lithium salt. **a** Schematic illustration of the negatively charged HNT (HNT^−^) treated by sodium hexametaphosphate (SHMP) [35]. **b** Schematic illustration of the positively charged halloysite (HNT^+^) treated by PDDA. **c** TEM image and the element mapping for HNT^+^. **d** XPS of N 1* s* for HNT^+^. **e** Zeta potential for the HNT, HNT^−^, and HNT^+^. **f** SEM images for HNT^+^. **g** Comparison of the binding energy of Li^+^ with PVDF, HNT inner surface, and outside charged surface of HNT^−^ and HNT^+^ by DFT calculations (Li^+^: purple; C: gray; F: cyan; H: white; O: red; N: blue; Si: yellow; and Al: rose red). **h** Comparison of the binding energy of TFSI^−^ with PVDF, HNT inner surface, and outside charged surface of HNT^−^ and HNT^+^ by DFT calculations

Pristine HNTs have a one-dimensional structure with a minor amount of negative charge on the surface and positive charge within the inner surface [37]. We used hexametaphosphate and poly(diallyldimethylammonium chloride) (PDDA) to chemically modify their outer surface and the surface charge characteristics (Fig. 2a, b**)**. As shown in Fig. 2c, the Si, O, and N elements from HNT surface, and the N element from PDD^+^ chain are detected on the HNT^+^. The X-ray photoelectron spectroscopy (XPS) spectrum in Fig. 2d shows that the signal of N 1* s* at the 402.0 eV is attributed to N^+^ in PDD^+^ [38]. This indicates the successful surface modification of HNTs by PDD^+^, which is fulfilled by the electrostatic interaction between PDD^+^ and HNT. The surface charge characteristics of the modified HNTs were measured by zeta potential testing. As shown in Figs. 2e and S2, compared to the pristine HNT (− 17.73 mV), the HNT^+^ shows a zeta potential of + 46.03 mV, indicating the positive surface charge characteristic from the quaternary ammonium groups on the HNT^+^. While the HNT^−^ shows a zeta potential of − 43.37 mV, due to adsorption of the orthophosphate from the SHMP, causing a stronger negative charge on the outer surface. Benefitting from the increased surface charges on the outer surface and thus the electrostatic repulsion, the dispersion quality of both HNTs^+^ and HNTs^−^ in organic solvent DMF is greatly improved compared to the agglomerated HNTs as shown in Figs. S1 and 2f. This is favorable to form uniform ion-conduction networks inside the polymer electrolytes.

To better understand the interface interactions, the adsorption energies of Li^+^/TFSI^−^ between HNT, HNT^−^, and HNT^+^ were calculated by using density functional theory (DFT). As shown in Fig. 2g, the interaction between Li^+^ and HNT^−^ (− 3.49 eV) is the strongest compared to that of HNT (− 3.38 eV), PVDF (− 2,49 eV), HNT inner surface (− 2.17 eV), and HNT^+^ (− 0.08 eV). As shown in Fig. 2h, an opposite trend is observed for the case of TFSI^−^. The binding energy of TFSI^−^ with different species obeys a decreasing trend of HNT inner surface (− 3.0 eV), HNT^+^ (− 2.35 eV), PVDF (− 1.43 eV), HNT^−^ (− 1.31 eV), and HNT (− 1.13 eV). These results reflect that the surface charge type of the HNTs generates a profound impact on the interface interactions. Both HNT^+^ and HNT^−^ are prone to adsorb TFSI^−^ anions in their positively charged inner surface. HNT^+^ exhibits remarkable interaction with TFSI^−^ than that of unmodified HNT. Likewise, the HNT^−^ prefers to adsorb Li^+^ due to the electrostatic interaction. Therefore, the HNTs with modified surface charge characteristics exhibit the potential for better dissociating the Li salt and changing the Li^+^-solvation structure, which will be discussed in detail later.

### Surface Charge-Enhanced Mechanical Interface in NCCPE

**Fig. 3:**
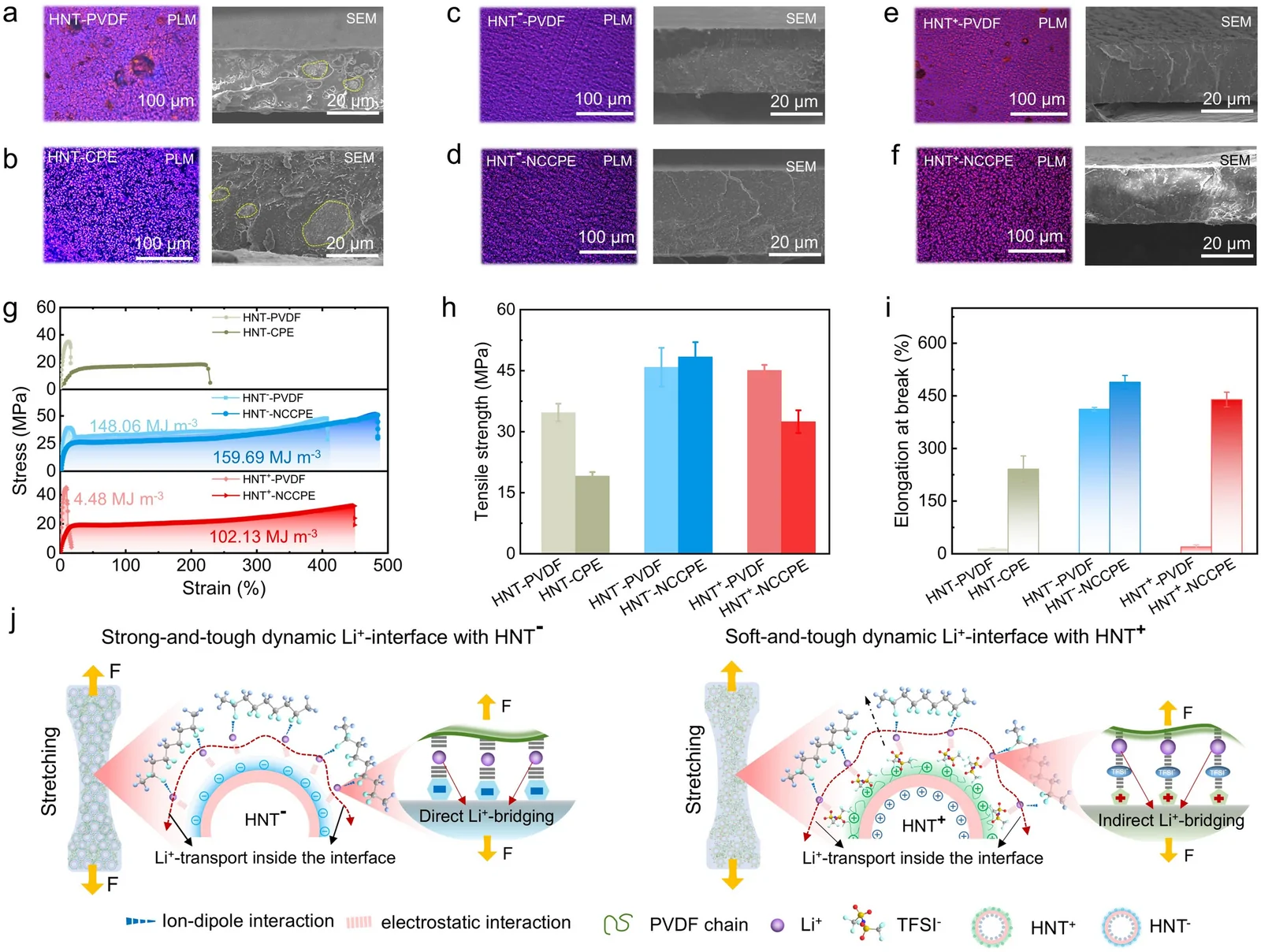
Studies on the mechanical reinforcement by the Li^+^-DI of the charged HNT in NCCPEs. Polarized light microscopy (PLM) images of the surface and SEM image of the cross-section for **a** HNT-PVDF, **b** HNT-PE, **c** HNT^−^-PVDF, **d** HNT^−^-NCCPE, **e** HNT^+^-PVDF, and **f** HNT^+^-NCCPE. **g** Strain–stress curves for PVDF-HNT nanocomposites with or without lithium salt. **h** Comparison of tensile strength of the HNT-CPE and NCCPEs with different surface charges as compared with the control samples without lithium salt. **i** Comparison of the elongation at break for the HNT-CPE and NCCPEs with different surface charges as compared with the control samples without lithium salt. **j** Schematic of possible different mechanical responses for the dynamic Li^+^-interfaces in HNT^−^-NCCPE and HNT^+^-NCCPE

The effects of surface charge characteristics of HNTs on their interface properties were first probed by using polarized light microscopy (PLM) and SEM imaging. As shown in Figs. 3a, b and S3, unmodified HNTs exhibit severe agglomeration in both PVDF and PVDF-LiTFSI matrices, which indicates the poor interface interaction between HNTs and PVDF. The dispersion quality is obviously improved by HNT^−^ and HNT^+^ in both PVDF and PVDF-LiTFSI matrices, as proved by the uniform distribution of nanotubes and the intimate interface contact (Fig. 3c–f). These findings are consistent with Fig. 2c showing that the charged HNTs have much improved dispersibility in the organic solvent. The addition of different types of charged HNTs also affects the crystallization behavior of PVDF. As shown in Figs. S3 and S4, adding the Li salt decreases the crystallization of PVDF, due to the ion–dipole interaction between PVDF and Li^+^. The crystallization of PVDF is further affected by the type of HNT nanofillers (Fig. S4). Compared to the electrolyte with HNT, the crystallinity of PVDF is further reduced by HNT^+^ and HNT^−^ whose crystallinity is 27.7% and 29.04%, respectively. However, we note that without Li salt, the effect of HNT nanofillers on the PVDF crystallization is insignificant as shown in Fig. S4b. This finding underlines the important role of Li salt in the interface interaction between PVDF and various HNT nanofillers, owing to the surface electrostatic interaction between HNT^−^/HNT^+^ and Li^+^/TFSI^−^. Besides, the decreased crystallinity of the polymer matrix will give rise to higher ionic conductivity and elongation at break.

Mechanical properties of various polymer composite films were measured to help understand how the surface charge characteristics influence the interface between PVDF and different HNTs. As shown in Fig. 3g–i, two types of polymer matrices were prepared, that is, pure PVDF and PVDF electrolyte (PVDF-LiTFSI). The tensile strength of HNT composited PVDF electrolyte (denoted as HNT-CPE) is 19.13 ± 0.94 MPa, which is lower than that of the HNT-PVDF (34.70 ± 2.18 MPa,), mainly caused by the suppressed crystallization of PVDF (also see Fig. S5 and Table S1). In addition, when PVDF is used as the matrix, the tensile strength of the HNT^−^ and HNT^+^ cases is 45.86 ± 4.76 and 45.08 ± 1.33 MPa, respectively, corresponding to the elongation at break of 411.89 ± 5.17% and 19.63 ± 5.87%. The overall tensile properties of the two cases are better than those of the pristine HNT case, which is attributed to the stronger adsorption of charged HNTs for ions and PVDF chains as well as better dispersion quality in the PVDF matrix. Meanwhile, the lower tensile properties of the HNT^+^-PVDF than the HNT^−^-PVDF is caused by the weak bonding strength. Interestingly, adding Li salt into PVDF significantly enhances the tensile properties for HNT^+^-PVDF and HNT^−^-PVDF, which is by contrast to the HNT-PVDF sample. Specifically, Li salt plays a more profound role in increasing the elongation at break for HNT^+^-NCCPE with an increasing of 22 times compared to HNT^+^-PVDF. In short, two key findings are revealed here. First, regardless of the matrix type, both tensile strength and elongation at break of samples with HNT^−^ are greater than those of HNT^+^. Second, the addition of Li salt makes more evident contribution to increasing the elongation at break of the HNT^+^-NCCPE compared to HNT^−^-PVDF.

To better understand the above findings, we propose a dynamic Li^+^-interface (Li^+^-DI) model as illustrated in Fig. 3j. For both HNT^−^-NCCPE and HNT^+^-NCCPE, the rich surface charges on the nanotube surface lead to stronger electrostatic adsorption toward ions. Based on the previous result in Fig. 2 that PVDF exhibits stronger interaction with Li^+^ than TFSI^−^, Li^+^ plays a more important role in enhancing the interface bonding strength between PVDF chains and various HNTs. For HNT^−^-NCCPE, the negative surface charges prefer to strongly adsorb Li^+^, while Li^+^ interacts with PVDF via Li^+^∙∙∙C-F bonding, forming a direct Li^+^-bridge at the PVDF/HNT^−^ interface. On the contrary, the HNT^+^ prefers to adsorb TFSI^−^ rather than Li^+^. Li^+^ is then adsorbed by TFSI^−^, leading to an indirect Li^+^-bridge forming between TFSI^−^ and PVDF. Therefore, when the HNT^−^-NCCPE is under mechanical deformation, the stronger bonding between PVDF chain and HNT^−^ via direct Li^+^-bridge leads to superior strength and toughness (159.69 MJ m^−3^). However, due to the indirect Li^+^-bridge that slightly weakens the bonding strength between PVDF chains and HNT^+^, the strength and toughness of HNT^+^-NCCPE are lower than those of HNT^−^-NCCPE. It is noted that the residual DMF also participates in the coordination with Li^+^ together with HNTs, anions, and PVDF as revealed by our previous study [35], but it is omitted here for highlighting the primary difference of ionic interaction with PVDF and HNT^+^/HNT^−^ at the Li^+^-DI. These findings emphasize that the type and strength of surface charges of HNTs are the key factors to the PVDF/HNT interface strength, which further affect the overall mechanical properties of the resultant composite electrolytes.

### Li^+^-Solvation Structure and Ion-Transport Mechanism of NCCPE

**Fig. 4:**
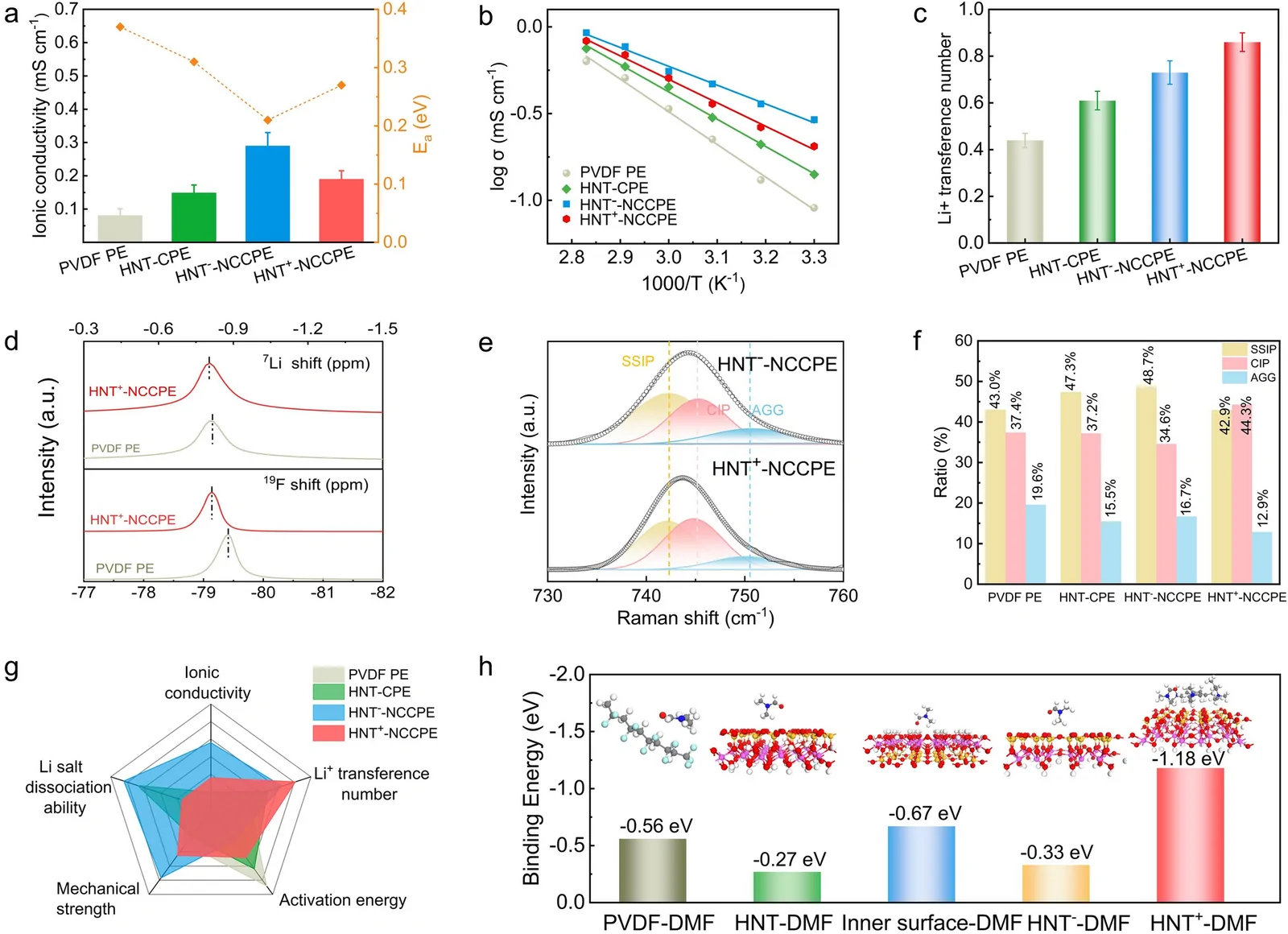
Experimental studies on the ion transport and solvation structures of the HNT-supported Li^+^-DI.** a** Ionic conductivity and active energy of the NCCPEs as compared with the conventional CPEs. **b** Arrhenius plots of the NCCPEs as compared with the conventional CPEs (the data for HNT^−^ are from Ref. [35]). **c** Comparison of the Li^+^-transference number of the NCCPEs with conventional CPEs. **d** Solid-state nuclear magnetic resonance (ss-NMR) spectra for ^7^Li and ^19^F. **e** Raman spectra of HNT^−^-NCCPE and HNT^+^-NCCPE, respectively. **f** Ion coordination state of the NCCPEs obtained from the Raman spectra results. **g** Radar diagram comparing the electrochemical performances of the NCCPEs with conventional CPEs. **h** Comparison of the binding energy of DMF with PVDF, HNT, inner surface, HNT^−^, or HNT^+^ from DFT calculation. Colors: Li^+^: purple; C: gray; F: cyan; H: white; O: red; N: blue; Si: yellow; and Al: rose red

To investigate the ion-transport behavior of different charged HNTs, the ionic conductivity of various composite electrolytes was measured at different temperatures. As shown in Fig. 4a, HNT^+^-NCCPE exhibits an ionic conductivity of 0.19 ± 0.025 mS cm^−1^ at room temperature, which is lower than that of HNT^−^-NCCPE (0.27 ± 0.035 mS cm^−1^**)** but higher than PVDF electrolyte and HNT-CPE (also see Fig. S6). To minimize the plasticizing effect of DMF residue on the electrolyte properties, we controlled the DMF content of each sample at a relatively low and similar level. The residual DMF content of the PVDF electrolyte, HNT-CPE, HNT^+^-NCCPE, and HNT^+^-NCCPE is 6.41, 6.18, 4.86, and 5.80 wt.%, respectively (Fig. S7). Therefore, the improved ionic conductivity of two NCCPEs is not solely attributed to the residual solvent. To gain a deeper understanding that how the charged HNTs control the ion-transport kinetics, the activation energies were calculated. As shown in Figs. 4b and S8, the Arrhenius plots show that the activation energy (${E}_{a}$) follows a decreasing trend of PVDF electrolyte (0.37 eV), HNT^+^-NCCPE (0.27 eV), and HNT^−^-NCCPE (0.21 eV) [35]. Additionally, the Li^+^-transference number follows an increasing trend of PVDF electrolyte (0.44 ± 0.03), HNT-CPE (0.61 ± 0.04), HNT^−^-NCCPE (0.73 ± 0.05), and HNT^+^-NCCPE (0.86 ± 0.04) as shown in Figs. 4c and S9. The positive-charge surface of HNT^+^ presents more anchored anion sites than HNT^−^ to improve the Li^+^ transference number, which can be proved by Zeta potential data. All these findings imply that the surface charge characteristics of HNTs strongly impact the ion-transport behavior of the composite electrolytes. The HNT^+^-NCCPE shows high Li^+^-transference number and good mechanical strength in comparison with recently reported CPEs (Table S2).

Since Li^+^-solvation structure is the fundamental factor to the ion-transport mechanism of electrolytes, we probe the Li^+^-solvation structure of electrolytes with various types of HNTs. Solid-state nuclear magnetic resonance (SSNMR) of the ^7^Li and ^19^F spectra was employed to analyze the TFSI^−^-Li^+^ ion pair interaction. As shown in Fig. 4d, the peak of the ^7^Li spectra is shifted to the downfield, indicating weakened Li^+^ and TFSI^−^ interaction in the HNT^+^-NCCPE. This result is in agreement with the ^19^F spectra [39]. Raman spectroscopy was further used to monitor the changes in the Li^+^-TFSI^−^ ionic interactions. As shown in Figs. 4e, f and S10a, b, the S–N-S band of TFSI^−^ exhibits three modes: free TFSI^−^ (SSIP, 740 cm^−1^), TFSI^−^ contact ion pairs with a single Li^+^ (CIP, 745 cm^−1^), and TFSI^−^ aggregated ion pairs with two or more Li^+^ (AGG, 750 cm^−1^) [40]. In detail, HNT^−^-NCCPE demonstrates a higher content of SSIP (48.7%) than that of HNT^+^-NCCPE (42.9%). This indicates that HNT^−^ has a better capability to dissociate Li salt due to its higher adsorption to Li^+^ (− 3.38 eV) compared to the adsorption between HNT^+^ and TFSI^−^ (− 2.35 eV) as revealed by the DFT calculation in Fig. 2g, h. In addition, more anions are involved in the Li^+^-solvation structure of HNT^+^-NCCPE, which is indicated by the domination of CIP (44.5%) and AGG (12.9%) of anions. This result is consistent with the DFT data showing that the stronger adsorption between HNT^+^ and TFSI^−^ causes the formation of TFSI^−^-rich solvation structure. Such a weak solvation structure leads to a relatively sluggish ion-transport kinetics, which will be discussed in detail later.

Based on the performance summary in Fig. 4g, HNT^−^-NCCPE has higher ionic conductivity (0.29 mS cm^−1^) and lower activation energy (0.21 eV) in comparison with HNT^+^-NCCPE (0.19 mS cm^−1^; 0.27 eV) and HNT-CPE (0.14 mS cm^−1^; 0.31 eV). This is because HNT^−^ has better Li salt dissociation to generate more charge carriers and a strong Li^+^-solvation structure to facilitate the Li^+^-movement [41]. HNT^−^-NCCPE also has a higher mechanical strength because of the stronger Li^+^-DI as revealed before. However, the Li^+^-transference numbers of HNT-CPE (0.61 ± 0.04) and HNT^−^-NCCPE (0.73 ± 0.05) are lower than that of HNT^+^-NCCPE (0.86 ± 0.04), because both outer and interior surfaces of HNT^+^ provide electrostatic adsorption to anions, while only interior surface of HNT^−^ can do. Since the existence of residual DMF solvent affects the Li^+^-solvation status via the dielectric screening and coordination competition effects, DFT calculations were further performed to calculate the binding energy between DMF and different HNTs. As shown in Fig. 4h, the binding energy between C=O group of DMF and HNT^+^ is − 1.18 eV, which is significantly higher than that of pristine HNT (− 0.27 eV), HNT^−^ (− 0.33 eV), and PVDF (− 0.56 eV). The strong adsorption between DMF and HNT^+^ makes the looser coordination between Li^+^ and DMF and allows TFSI^−^ to competitively participate in the solvation structure. Meanwhile, HNT^+^ effectively captures DMF solvent to resist its side reactions and decomposition on the Li-metal anode. As a result, the electrochemical window of HNT^+^-NCCPE is extended to 4.80 V (Fig. S11), which is broader than that of HNT-NCCPE (4.65 V).

**Fig. 5:**
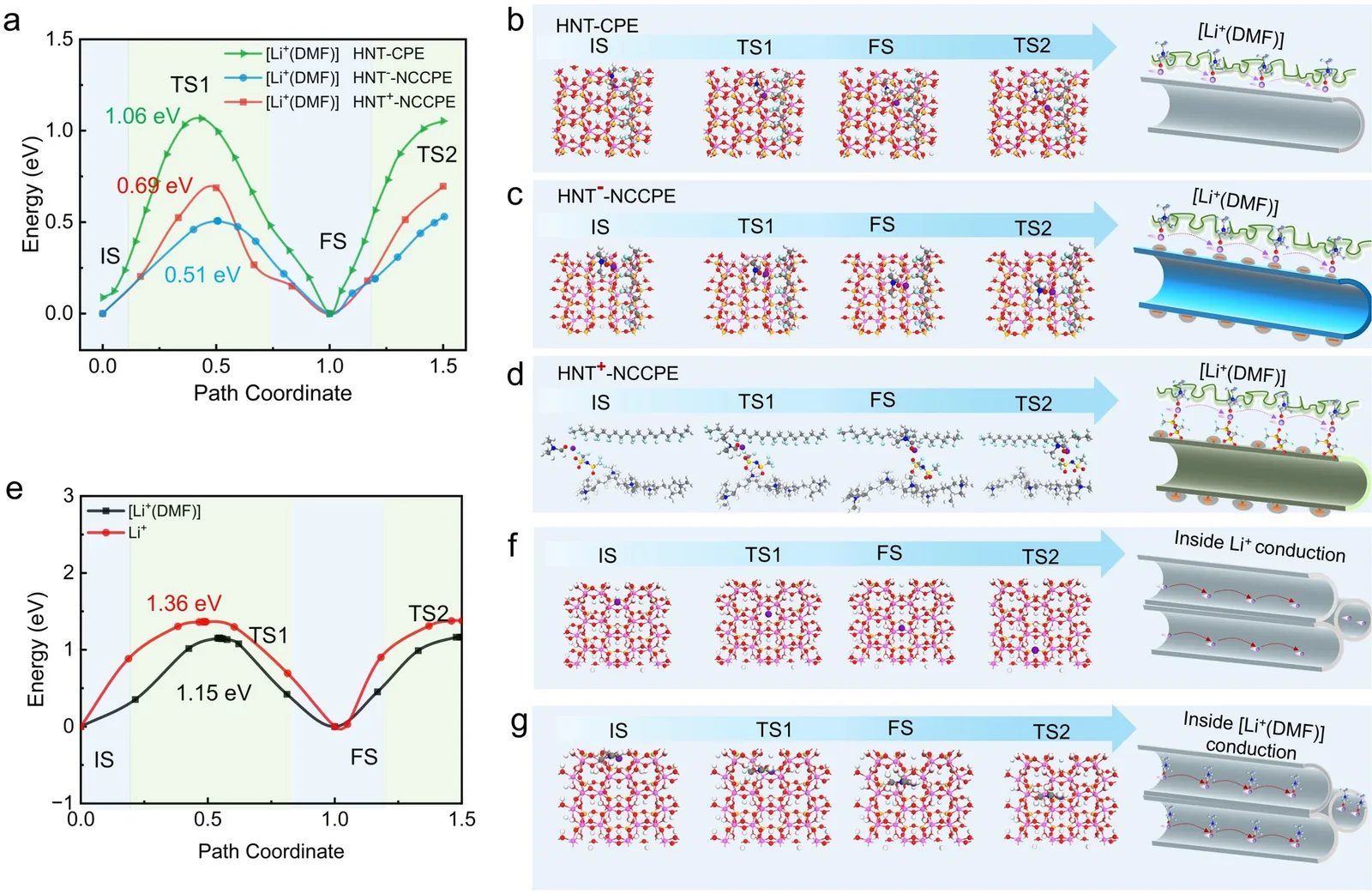
Simulation studies of the effects of surface charge characteristics on the lithium-ion transport dynamics inside the Li^+^-DI.** a** Migration energy barriers for [Li^+^(DMF)] at the Li^+^-DI between PVDF and charged HNT along with the axial pathways. **b**-**d** Simulation snapshots of the [Li^+^(DMF)] migration behaviors inside the PVDF/HNT, PVDF/HNT^−^, and PVDF-HNT^+^ interfaces, respectively. **e** Migration energy barriers for [Li^+^(DMF)] and Li^+^ inside the inner surface of the charged HNT along the axial pathways. **f, g** Simulation snapshots of the Li^+^-hopping and [Li^+^(DMF)] hopping behaviors inside inner surface along the axial pathways, respectively. The initial, transition, and final states are abbreviated as IS, TS, and FS, respectively. Colors: Li^+^: purple; C: gray; F: cyan; H: white; O: red; N: blue; Si: yellow; and Al: rose red

To gain a comprehensive understanding of the ion-transport behavior of the HNT-supported Li^+^-DI, Transition-State-Density-Functional-Theory (TS-DFT) calculations were conducted to investigate the influence of the surface charge characteristics of HNTs on Li^+^-transport energy barrier. Since HNTs have inner and outside surfaces, the ionic migration energy barriers of [Li^+^(DMF)] inside the PVDF/HNT interface, that is the Li^+^-DI, along the axial was investigated first (Fig. 5a). Based on the calculation, the [Li^+^(DMF)] migration energy barriers of the HNTs^+^-supported Li^+^-DI and the HNTs^−^-supported Li^+^-DI are 0.69 and 0.51 eV, respectively, which are significantly lower than that of the PVDF/HNT interface (1.06 eV). These results are consistent with the Arrhenius energy calculations as previously discussed. The corresponding simulation snapshots of the [Li^+^(DMF)] migration behaviors and the schematic diagram are shown in Fig. 5b–d. From the simulation studies, one can find that the Li^+^ is basically coupled with the absorbed solvent molecule of DMF, which form [Li^+^(DMF)] complex. The surface charges of HNTs can reduce the energy barrier for the hopping of [Li^+^(DMF)], but not the distance for each hopping. Furthermore, a negative-charged surface is more effective to reduce the hopping energy barrier which may indicate some interesting physics behind the hopping. The migration energy barrier of Li^+^ and [Li^+^(DMF)] complex along the axial direction in the inner surface of HNTs has also been calculated as shown in Fig. 5e. The [Li^+^(DMF)] has an energy barrier of 1.15 eV, which is lower than that of Li^+^ (1.36 eV). This indicates that Li^+^ undergoes solvent-assisted rapid transport within the nanochannels of HNTs. The corresponding simulation snapshots at different transition states and the schematic diagram are shown in Fig. 5f, g. From the above simulation studies, we can find that the surface charge characteristics of HNTs play a critical role in controlling the Li^+^-transport kinetics at the Li^+^-DI. In specific, the HNT^−^-supported Li^+^-DI has lower migration energy barrier than that of the HNT^+^-supported counterpart. At the same time, the inner surface of HNTs can also absorb DMF solvent and form conduction pathway, but its energy barrier for Li^+^-hopping is notably higher than that of Li^+^-DI. These results agree with the above studies, which reveal that the HNT^−^-supported Li^+^-DI exhibits more free Li^+^ ions and a strong Li^+^-solvation structure while the HNT^+^-supported Li^+^-DI generates less free Li^+^ ions and anion-rich Li^+^-solvation structure. The former imparts faster ion-transport kinetics or lower ion-hopping energy barrier compared to the latter.

### Electrode/Electrolyte Interface Stability and Solid-State Battery Performance

**Fig. 6:**
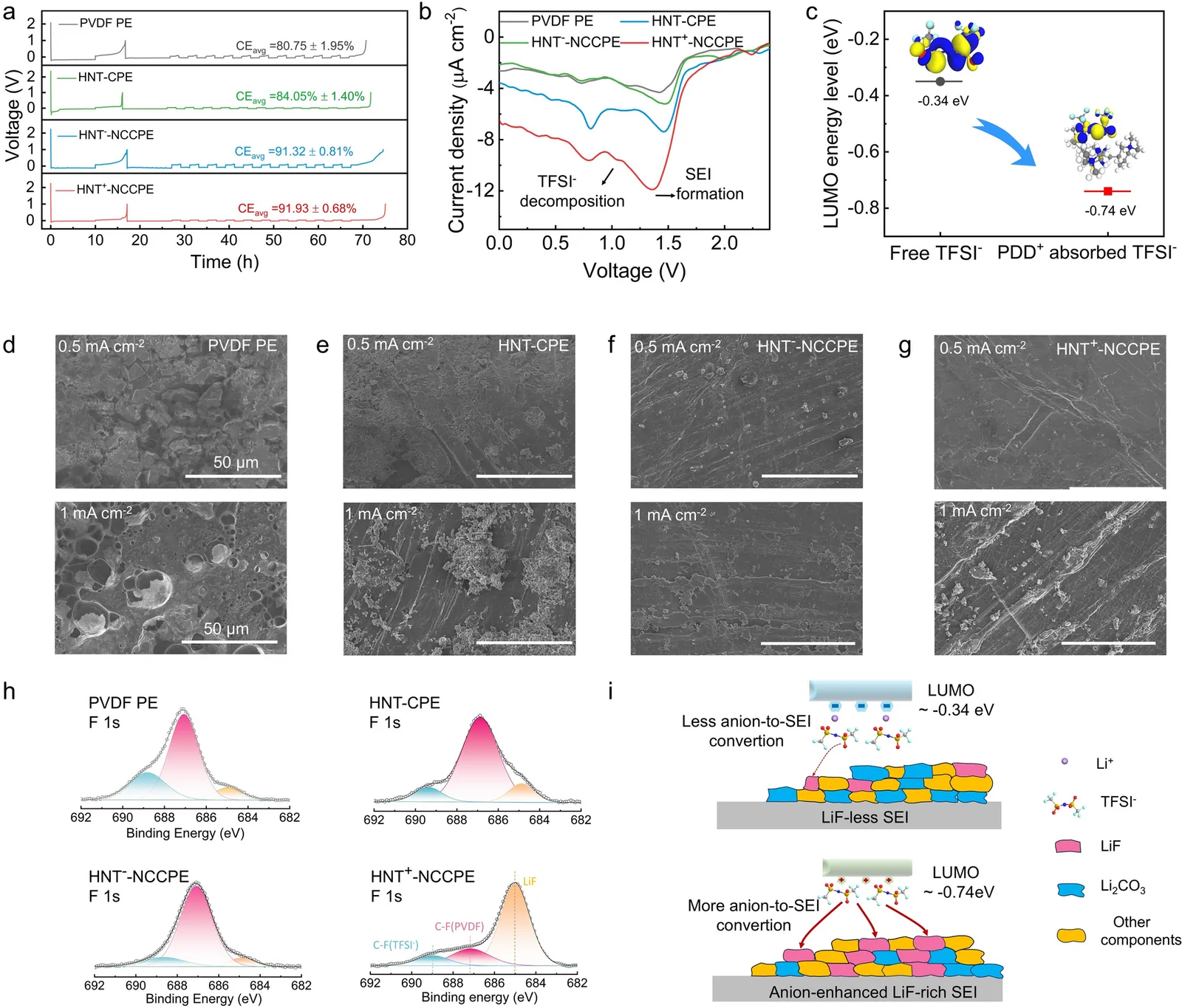
Analysis of the characteristics of the solid–electrolyte interphase (SEI) derived from the NCCPEs.** a** Coulombic efficiency of the Li|NCCPEs|Cu cells as compared with the control samples at a current density of 0.2 mA cm^−2^. **b** Cyclic voltammetry curves of Li|NCCPEs|Cu cells at a scan rate of 0.5 mV s^−1^ from 2.5 to 0 V. **c** Comparison of the LUMO energy level of the free TFSI^−^ and HNT^+^-adsorbed TFSI^−^. **d-g** SEM images of the Li-metal anode disassembled from Li||Li symmetric cells after 30 cycles at an areal capacity of 0.5 mAh cm^−2^ at different current densities (0.5 and 1 mA cm^−2^) for pure PVDF-based PE, PVDF/HNT-based CPE, PVDF/HNT^−^-based NCCPE, and PVDF/HNT^+^-based NCCPE, respectively. **h** XPS curves of F1s of the cycled Li-anode in Li||Li cells with pure PVDF-based PE, PVDF/HNT-based CPE, PVDF/HNT^−^-based NCCPE, and PVDF/HNT^+^-based NCCPE. **i** Scheme illustration of the effects of HNT surface charges on the formation of SEI at Li-metal surface

The Li deposition and stripping behavior and electrochemical stability of the electrode/electrolyte interface were investigated in Li||Cu cells. Figure 6a shows the Li plating/stripping curves of Li||Cu cells with various electrolytes. The average Coulombic efficiency (CE) during Li plating/stripping was quantified according to modified Aurbach method [42, 43]. As noted in Fig. 6a, the average CE of HNT^+^-NCCPE is 91.4%, which is higher than that of HNT^−^-NCCPE (90.75%), HNT-CPE (85%), and PVDF electrolyte (79.4%). The result indicates that the Li plating/stripping process occurs smoothly in the HNT^+^-NCCPE cell, and there is minimal loss of Li from the deposited Cu current collector. The cyclic voltammetry (CV) curves were recorded from 2.5 to 0 V to help understand the interface reaction during Li plating/stripping process (Fig. 6b). For all cells, the broad peak at about 1.45 V is attributed to the SEI formation [44]. We note that an additional peak at 1.15 V appears for the HNT^+^-NCCPE, which is due to the reduction of TFSI^−^ [45]. This finding implies a more intensive decomposition of TFSI^−^ on the Li metal for the HNT^+^-NCCPE cell. To better understand the underlying mechanism, the lowest unoccupied molecular orbitals (LUMO) of TFSI^−^ and charge-adsorbed TFSI^−^ by PDD^+^ were investigated by DFT calculations. As shown in Fig. 6c, the LUMO of PDD^+^-adsorbed TFSI^−^ decreases to -0.78 eV compared to the free TFSI^−^ (− 0.34 eV), which suggests that the electron-accepting capability of TFSI^−^ is enhanced to facilitate the decomposition of TFSI^−^. Therefore, HNT^+^-NCCPE facilitates the decomposition of TFSI^−^ on Li-metal anode to form LiF component, which effectively enhances the interface stability and cycling stability of Li-metal anode as revealed in Fig. 6a.

Figure 6d–g shows the surface morphology of cycled Li metal after 30 cycles at different current densities of 0.5 mA cm^−2^ or 1 mA cm^−2^. As shown in Fig. 6d, e, uneven Li deposits and rough surface are observed for the PVDF electrolyte and HNT-CPE samples (the original Li-metal morphology is shown in **Fig. S12**). In contrast, the surface of the Li metal with HNT^−^-NCCPE remains relatively flat even at 1 mA cm^−2^ (Fig. 6f). Notably, the Li metal with HNT^+^-NCCPE exhibits a much smoother and uniform surface with no obvious dendrite growth or dead Li deposits even at a higher current rate of 1 mA cm⁻^2^ (Fig. 6g). These results demonstrate that HNT^+^-NCCPE efficiently promotes uniform Li flux and stable Li plating/stripping process. XPS was conducted to probe the surface chemistry of cycled Li-metal anodes. In the C 1* s* spectrum, the peak at 284.8 eV is attributed to C–C, while the peak at 286.2 eV is assigned to C-H. The peak at 288.8 eV is assigned to the carbonyl group of DMF and C-F of PVDF. The peak at 290.3 eV results from the C-F peak of PVDF (Fig. S13). In the F 1* s* spectra, the peak at 685.0 eV corresponds to inorganic LiF, while the peak at 687.1 eV is assigned to organic C–F originating from PVDF. Additionally, the peak at 689.01 eV is attributed to the C–F bond in TFSI^−^ species (Fig. 6h) [26, 35, 46]. According to the peak-area fitting results, the LiF content in the SEI is greatly improved by the HNT^+^-NCCPE compared to HNT^−^-NCCPE, HNT-CPE, and PVDF electrolyte. Therefore, as illustrated in Fig. 6i, more anions participate in the SEI formation for HNT^+^-NCCPE to enrich the LiF component due to its anion-rich Li^+^-solvation structure, in comparison with HNT^−^-NCCPE. The richer LiF component is beneficial to the mechanical strength of the SEI layer, which helps resist the SEI breakage and improves the stability of Li metal during cycling.

**Fig. 7:**
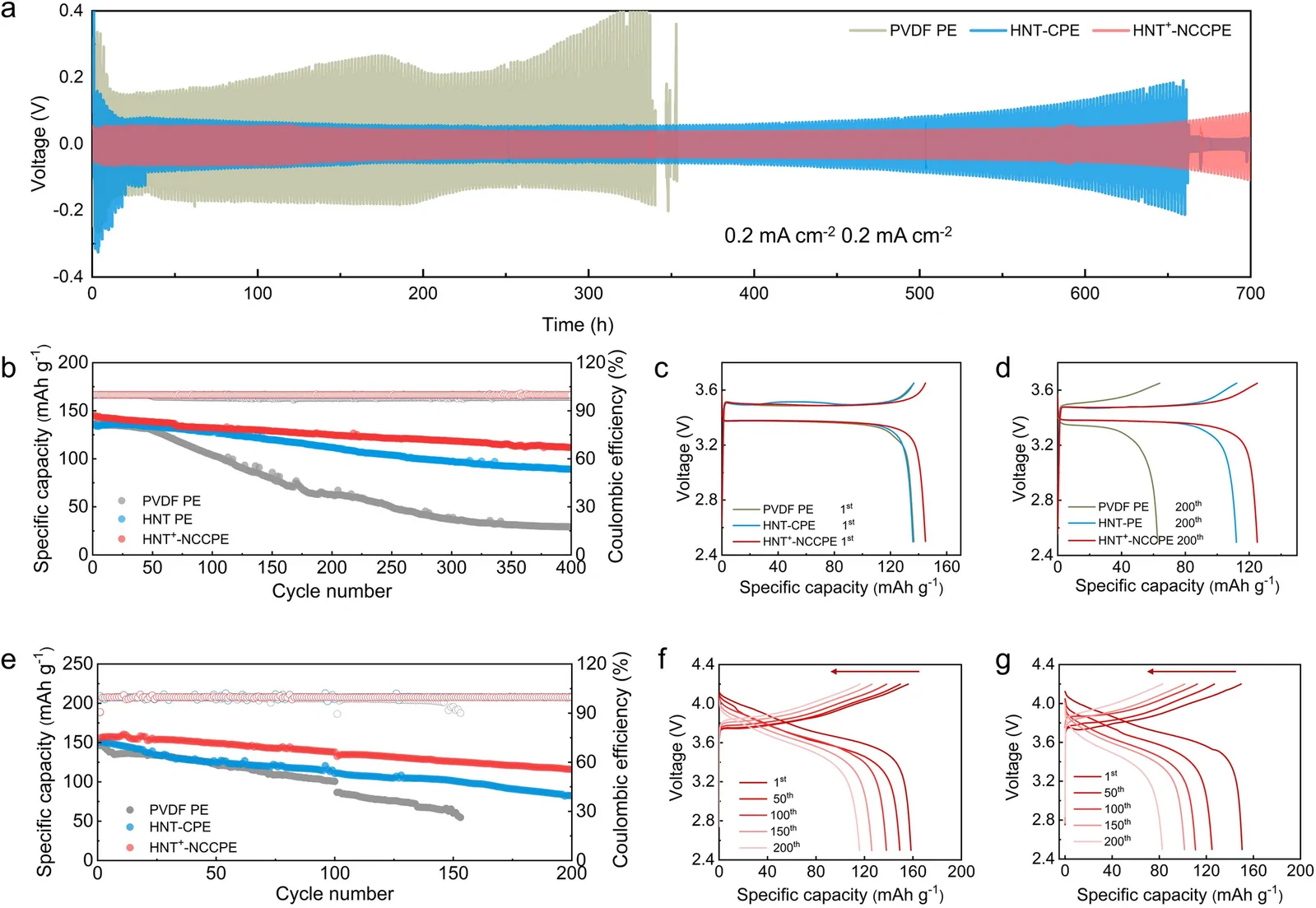
Electrochemical performance of NCCPE-based ASSLMB. **a** Galvanostatic cycling curves of Li||Li cells with HNT^+^-NCCPE at a current density of 0.2 mA cm^−2^. **b** Cycling stability of the Li|HNT^+^-NCCPE|LFP half-cell as compared with other solid polymer electrolytes. **c** The corresponding charge–discharge voltage profiles of Li||LFP cell at the 1st cycle. **d** The corresponding charge–discharge voltage profiles of Li||LFP cell at the 200th cycle. **e** Cycling stability of the Li| HNT^+^-NCCPE |NCM811 half-cell as compared with other solid polymer electrolytes. **f** The corresponding charge–discharge voltage profiles of Li|HNT^+^-NCCPE|NCM811 at different cycle numbers. **g** The corresponding charge–discharge voltage profiles of Li|HNT-CPE|NCM811 cell at different cycle numbers

Furthermore, Li||Li symmetrical cells were tested to study the long-term interface stability. The cell with HNT^+^-NCCPE stably cycles for over 700 h at 0.2 mA cm^−2^ with a stable polarization voltage of 36 mV (Fig. 7a). In contrast, the cell with HNT-CPE exhibits a much higher polarization voltage of 56 mV and gradual polarization increase after about 650 h. The performance degradation stems from the lower ionic conductivity of HNT-CPE and side reaction between DMF and Li metal, which exacerbate interfacial instability and polarization voltage amplification. Meanwhile, the robust LiF-rich SEI layer of the HNT^+^-NCCPE cell is beneficial to maintain stable electrolyte/Li-metal interface and reduce the formation of dead Li deposits, which decreases the interfacial resistance effectively. When increasing the current density to 0.25 mA cm^−2^, the cell with HNT^+^-NCCPE shows stable cycling performance up to 400 h, but the cells with PVDF electrolyte or HNT-CPE undergo short-circuiting after 120 and 260 h, respectively (Fig. S14). The critical current density (CCD) of the cell with HNT^+^-NCCPE was evaluated to assess its maximum current. As shown in Fig. S15, the cell exhibits stable cycling and can achieve a CCD up to 1 mA cm^−2^ due to its fast Li^+^ transport and the stability of the electrochemical interface. The above findings indicate that HNT^+^-NCCPE is effective in stabilizing the Li-metal anode, owing to its overall advantages in ionic conductivity, Li^+^-transference number, and solvent capture ability. Particularly, the high Li^+^-transference number of the HNT^+^-NCCPE plays the important role in suppressing the concentration polarization and dendrite growth, which effectively enhances the cycling stability and reduces the polarization voltage of the symmetrical cell. These results demonstrate that the HNT^+^-NCCPE exhibits excellent electrochemical stability against Li-metal anode and superior electrochemical performance compared with other recently reported CPEs (Table S3).

Figure 7b–d shows the long-term cycling performance of the Li||LFP cells at 2.5–3.65 V. The cell with HNT^+^-NCCPE delivers an initial discharge specific capacity of 144.9 mAh g^−1^ with a capacity retention of 78.6% at 0.5 C after 400 cycles (Fig. S16a). In comparison, the capacity retention of the cells with HNT-CPE and PVDF electrolyte is 65.0% and 21.0%, respectively (Fig. S16b, c). Additionally, the C-rate performance of the cell with HNT^+^-NCCPE is superior to that of HNT-CPE and PVDF electrolyte. When cycling at 0.1, 0.2, 0.5, 1, and 2 C, the cell with HNT^+^-NCCPE delivers the highest discharge capacities of 156.1, 150.6, 137.1, 119.3, and 88.4 mAh g^−1^, respectively (Fig. S17). Voltage profiles also show stable charge–discharge plateaus at different current rates (Fig. S18a–c). The improved C-rate performance further confirms the combined effect from HNT^+^-NCCPE, including high ionic conductivity, Li^+^-transference number, and electrochemical interface stability.

To assess the practical potential of such composite electrolytes, the cycling stability of Li||NCM811 cells was also evaluated. As shown in Fig. 7e, f, the cell with HNT^+^-NCCPE delivers an initial specific capacity of 157.1 mAh g^−1^ and exhibits a capacity retention of 74.4% after 200 cycles at 0.5 C. In sharp contrast, the cell with HNT-CPE exhibits a discharge capacity of 150.4 mAh g^−1^ with a capacity retention of 54.9% after 200 cycles. The cell with PVDF electrolyte only shows a capacity retention of 12.3% (Fig. S19). The batteries with HNT^+^-NCCPE exhibit comparable long-term cycling performance compared with recently reported PVDF-based and other composite solid-state electrolytes (Table S4). The high-voltage compatibility was measured by increasing the cut-off voltage to 4.4 V. As shown in Figs. S20 and S21, the cell with HNT^+^-NCCPE delivers a specific capacity of 193.1 mAh g^−1^ with a capacity retention of 76.68% after 100 cycles at 0.5 C, indicating a good high-voltage compatibility. The resistance of the cells with different electrolytes was measured in Fig. S22a–c. The charge-transfer resistance (*R*_ct_) of the cells with HNT-CPE and HNT^+^-NCCPE gradually decreases during cycling. The *R*_ct_ of the fresh HNT^+^-NCCPE cell is 227 Ω, which decreases to 84 Ω after 100 cycles. In comparison, HNT^+^-NCCPE shows higher CE, CCD, capacity retention, and lower *R*_ct_ (Table S5). The gradual decrease of *R*_ct_ during cycling may be attributed to the activation of the interphase and the formation of a stable ion-conducting layer.

## Conclusion

In conclusion, this study has revealed the roles of surface charges of HNTs in regulating the mechanical and electrochemical interfaces of nano-charged composite polymer electrolytes (NCCPEs). The charged HNTs can absorb the ions of Li salt and residual solvent to form a Li^+^-dynamic interface (Li^+^-DI), which can be adjusted by the surface charge characteristics of HNTs. Results show that the Li^+^-DI plays a pivotal role in controlling ion-conduction and ion-bridge, which is the fundamental factor for enhancing the mechanical and electrochemical interfaces of the NCCPEs. Specifically, the positively charged HNTs^+^ can anchor the anion of Li salt, achieving a high Li^+^-transference number. Meanwhile, the capture of DMF by HNTs^+^ weakens the coordination strength of Li^+^ with the C=O group of DMF and generates the anion-rich Li^+^-solvation structure. This HNTs^+^-controlled Li^+^-solvation structure forms a unique Li^+^-DI with fast ion-conduction and soft-and-tough mechanical response, which improves the toughness by over 2000% compared with the control without the Li^+^-DI. Meanwhile, the simulation and experimental results all show that the surface charge of HNT^+^ can reduce the LUMO energy of TFSI^−^ (− 0.74 eV) and promote its decomposition to form a stable LiF-rich SEI. Benefitting from these properties, the LFP||Li cell with HNT^+^-NCCPE delivers a superior cycling stability with a high Coulombic efficiency of 99.96% and capacity of 78.6% after 400 cycles at 0.5 C. This study provides a deep insight into how the surface charge of nanofillers affects the mechanical and electrochemical interfaces of composite polymer electrolytes, which may lay the foundation for the design and fabrication of advanced NCCPEs for solid-state Li-metal batteries.

## Supplementary Information

Below is the link to the electronic supplementary material.

Supplementary file1 (DOCX 3614 KB)
