# Supplementary material for: Lithium-Ion Dynamic Interface Engineering of Nano-Charged Composite Polymer Electrolytes for Solid-State Lithium-Metal Batteries
Source: Nanomicro Lett. 2025 Aug 29;18:46. doi: 10.1007/s40820-025-01899-7 (PMC12397479; doi:10.1007/s40820-025-01899-7)
Supplement: Supplementary file 1 — Supplementary file1 (DOCX 3614 KB) [file 40820_2025_1899_MOESM1_ESM.docx]

Supporting Information for

**Lithium-Ion Dynamic Interface Engineering of Nano-Charged Composite Polymer Electrolytes for Solid-State Lithium-Metal Batteries**

Shanshan Lv^1^, Jingwen Wang^1^, Yuanming Zhai^2^, Yu Chen^1^, Jiarui Yang^1^, Zhiwei Zhu^1^, Rui Peng^1^, Xuewei Fu^1^*, Wei Yang^1^ and Yu Wang^1^*

^1^ College of Polymer Science and Engineering, National Key Laboratory of Advanced Polymer Materials, Sichuan University, Chengdu 610065, P. R. China

^2^ Analytical & Testing Center, Sichuan University, Chengdu 610065, P. R. China

*Corresponding authors. E-mail: [xuewei.fu@scu.edu.cn](mailto:xuewei.fu@scu.edu.cn) (Xuewei Fu); [yu.wang3@scu.edu.cn](mailto:yu.wang3@scu.edu.cn) (Yu Wang)

**Supplementary Figures and Tables**

**
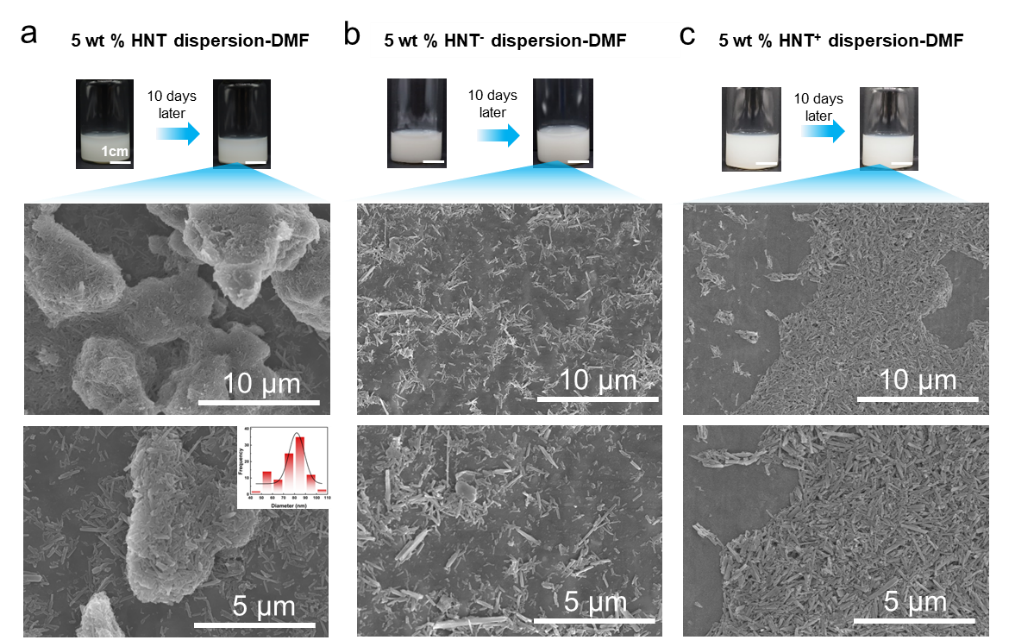
**

**Fig. S1** SEM images of (**a**) HNT, (**b**) HNT^-^and (**c**) HNTs^+^


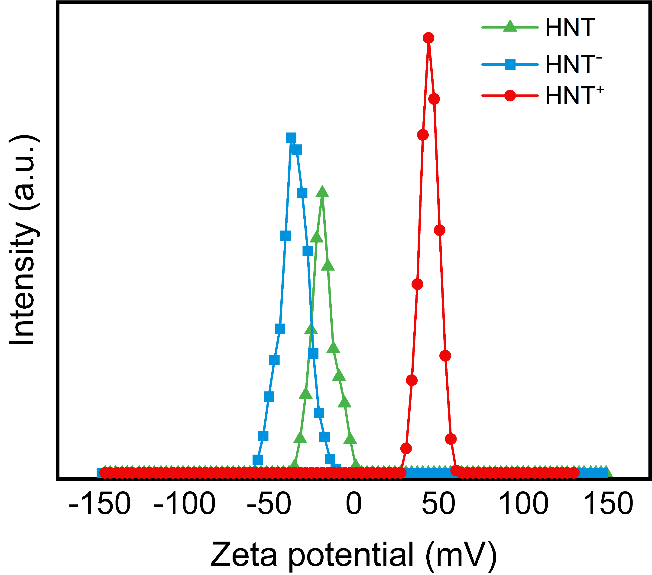


**Fig. S2** Zeta potential curves of HNTs, HNTs^-^ and HNTs^+^


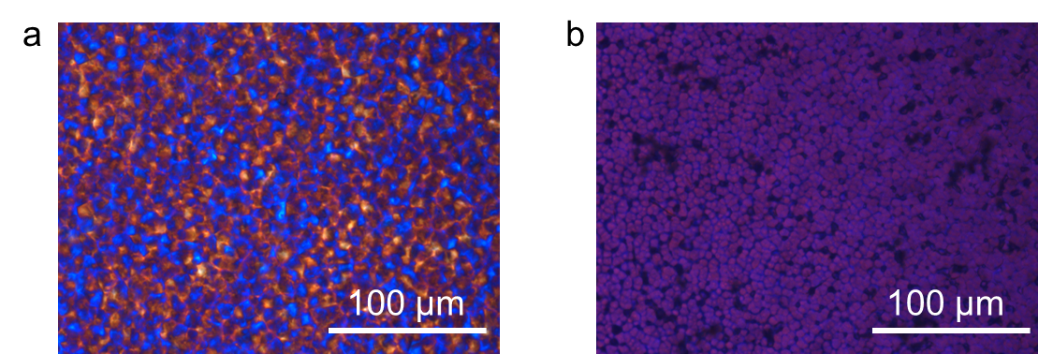


**Fig. S3** Polarized optical microscopy images of (**a**) PVDF and (**b**) PVDF electrolyte


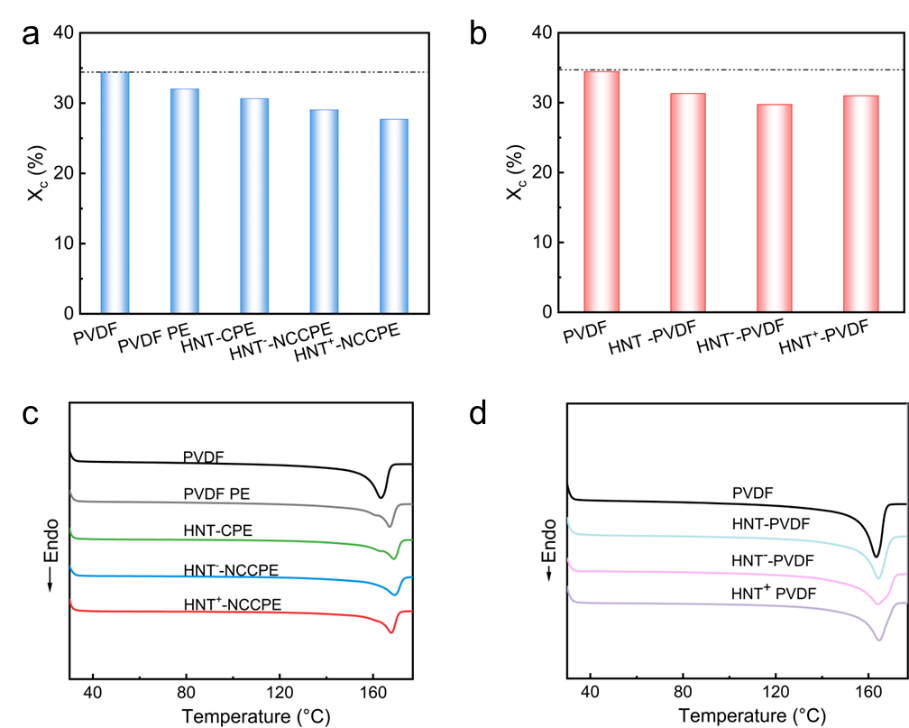


**Fig. S4** (**a**) Crystallinity degree of PVDF-based electrolytes. (**b**) Crystallinity degree of PVDF-based films without LiTFSI. DSC curves of (**c**) PVDF-based electrolytes and (**d**) PVDF-based films without LiTFSI


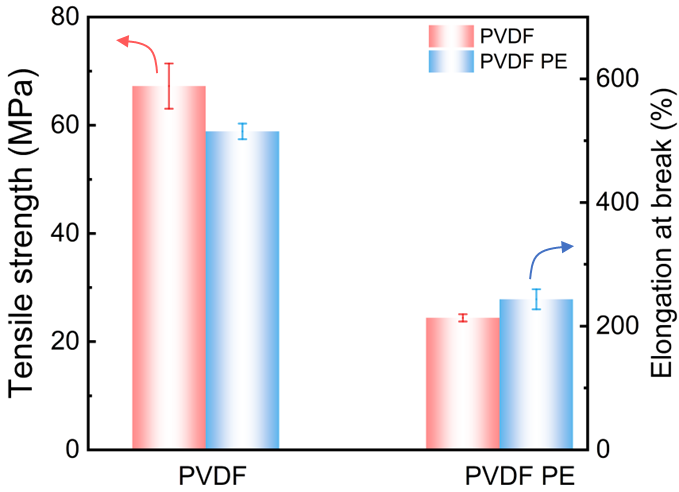


**Fig. S5** Tensile strength and elongation at break of PVDF and PVDF electrolyte


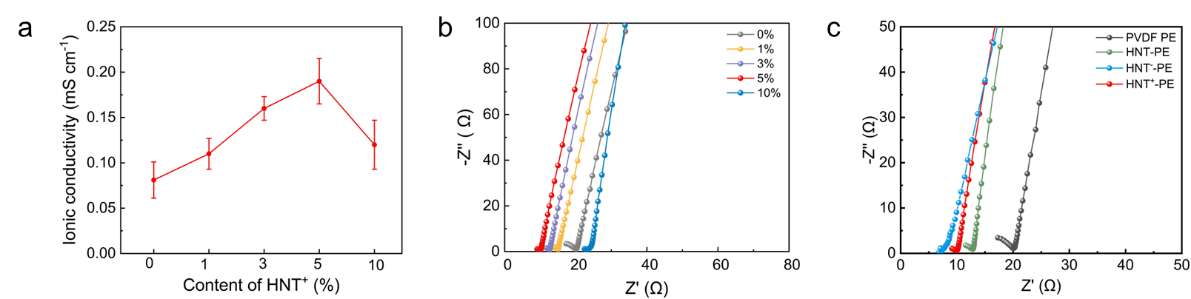


**Fig. S6** (**a**) Ionic conductivity of HNT^+^-NCCPE with different contents of HNTs**^+^**. (**b**) EIS spectra of HNT^+^-NCCPE with different contents of HNTs**^+^**. (**c**) EIS spectra of different electrolytes


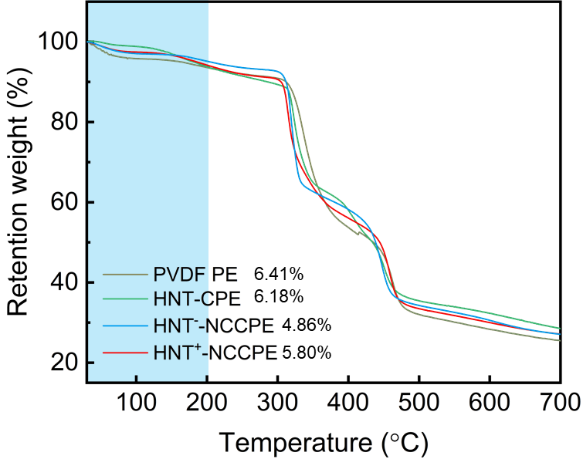


**Fig. S7** TGA curves of PVDF-based electrolytes


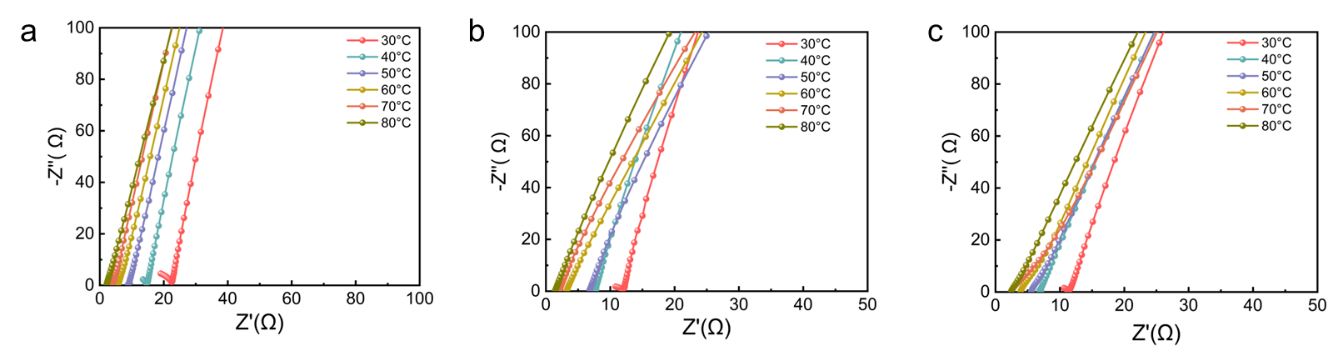


**Fig. S8** EIS spectra of (**a**) PVDF electrolyte, (**b**) HNT-CPE, and (**c**) HNT^+^-NCCPE under varying temperatures


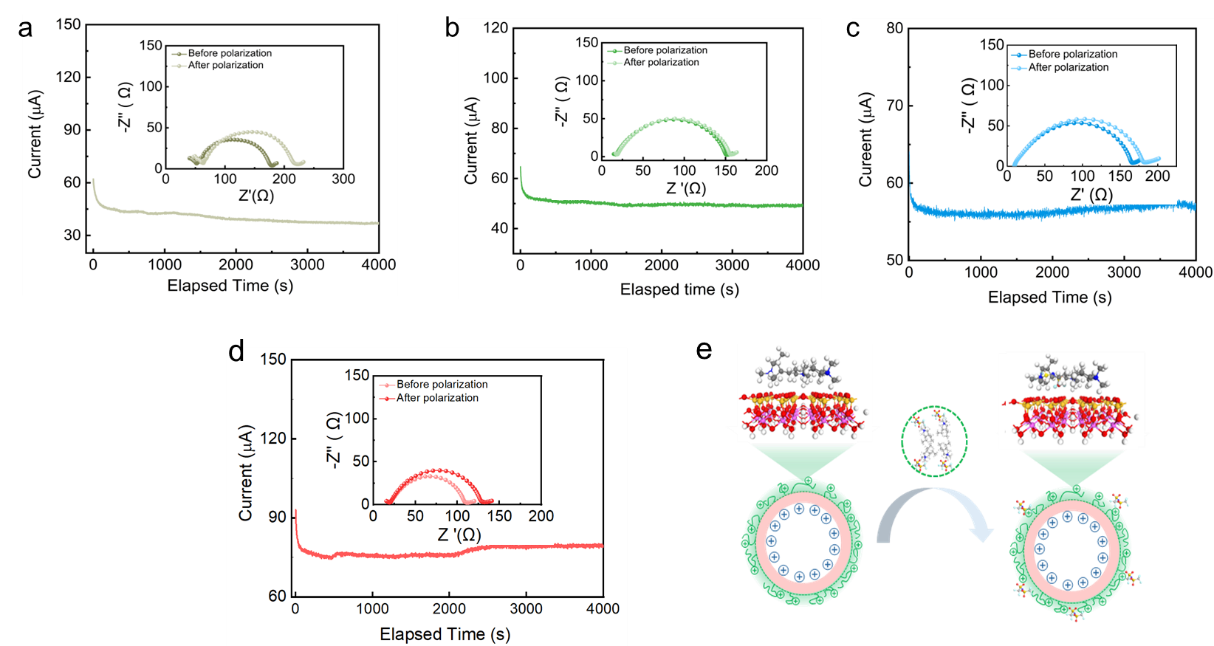


**Fig. S9** Current-time curves and EIS spectra before and after the polarization of Li||Li symmetric cells with (**a**) PVDF electrolyte, (**b**) HNT-CPE, (**c**) HNT^-^-NCCPE, and (**d**) HNT**^+^**-NCCPE. (**e**) Illustration of the anchored TFSI^-^ by HNT**^+^**


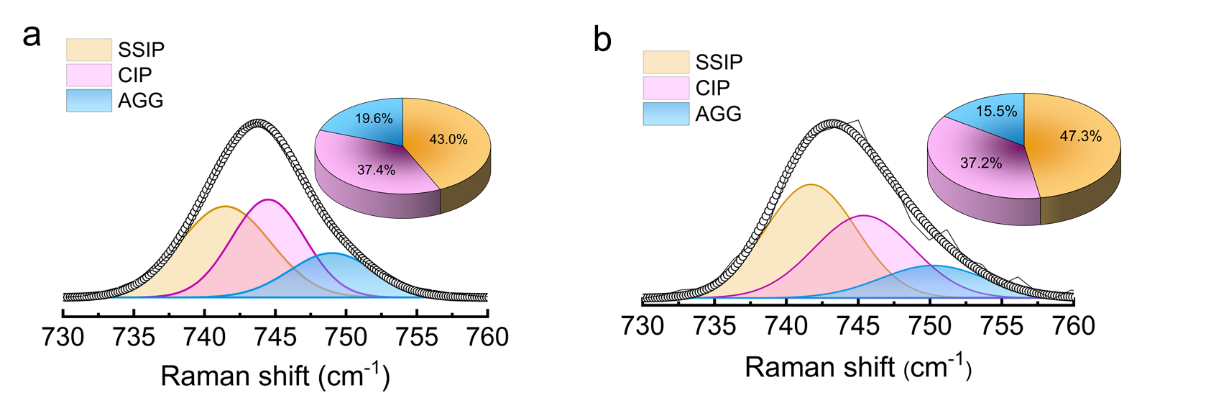


**Fig. S10** Raman spectra of (**a**) PVDF electrolyte and (**b**) HNT-CPE


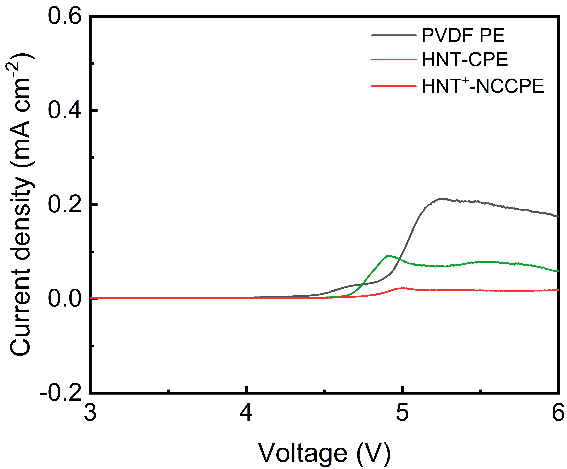


**Fig. S11** LSV curves of various PVDF-based electrolytes


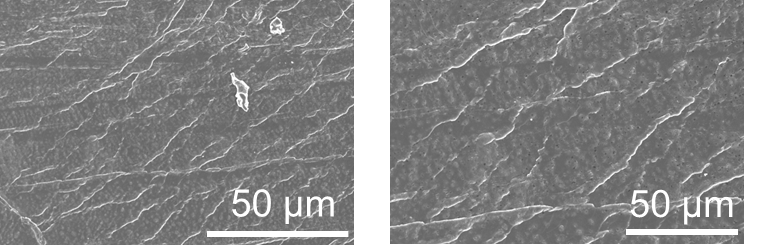


**Fig. S12** SEM images of fresh lithium metal


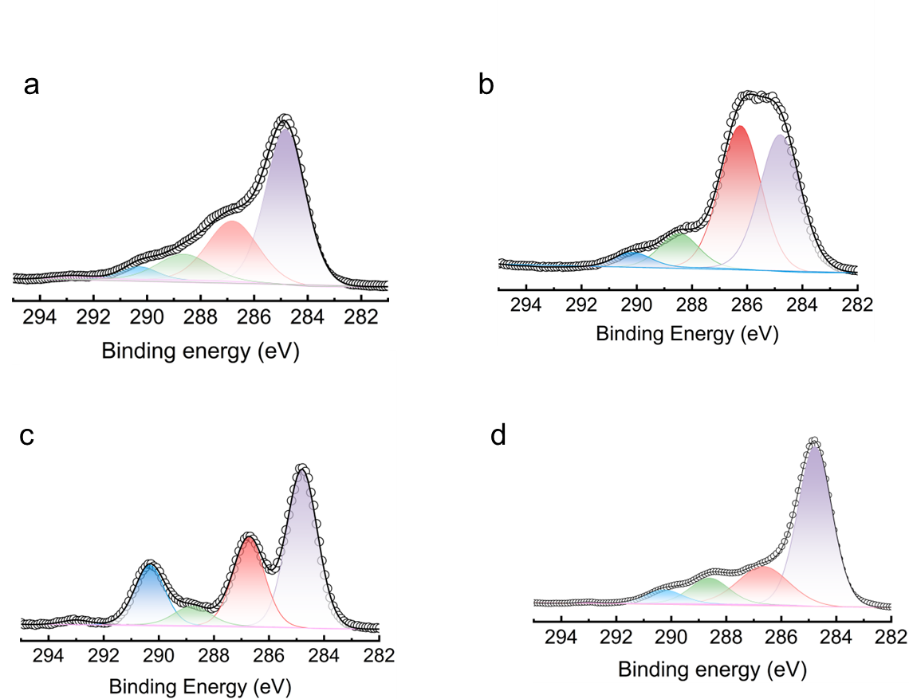


**Fig. S13** C1s spectra of cycled lithium metal with (**a**) PVDF electrolyte, (**b**) HNT-CPE, (**c**) HNT^-^-NCCPE and (**d**) HNT^+^-NCCPE

**
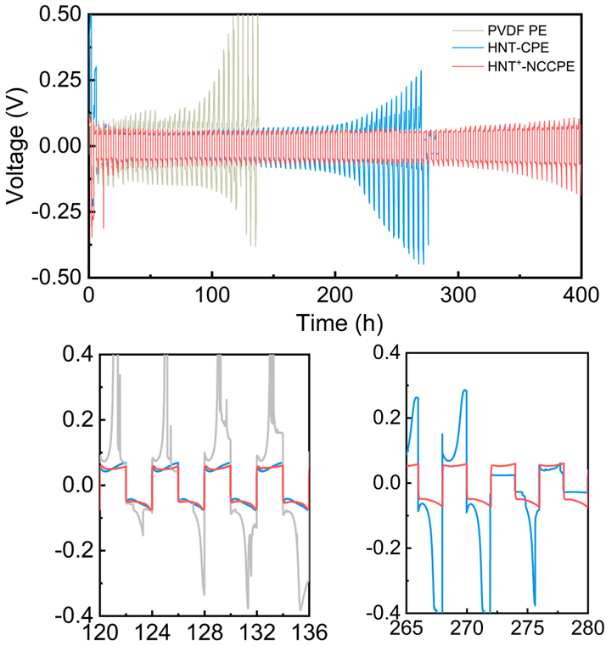
**

**Fig. S14** Galvanostatic cycling profiles of Li||Li symmetric cells with PVDF electrolyte, HNT-CPE and HNT**^+^**-NCCPE


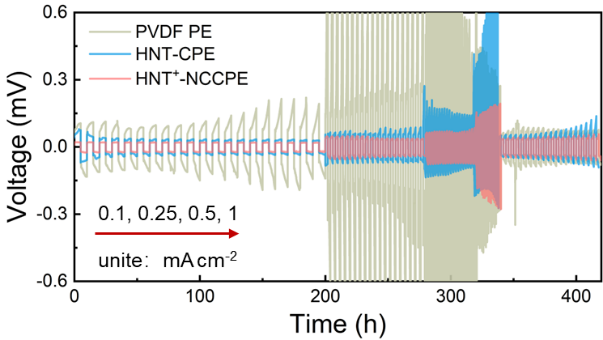


**Fig. S15** Critical current density (CCD) profiles of Li||Li symmetric cells with PVDF electrolyte, HNT-CPE, and HNT^+^-NCCPE


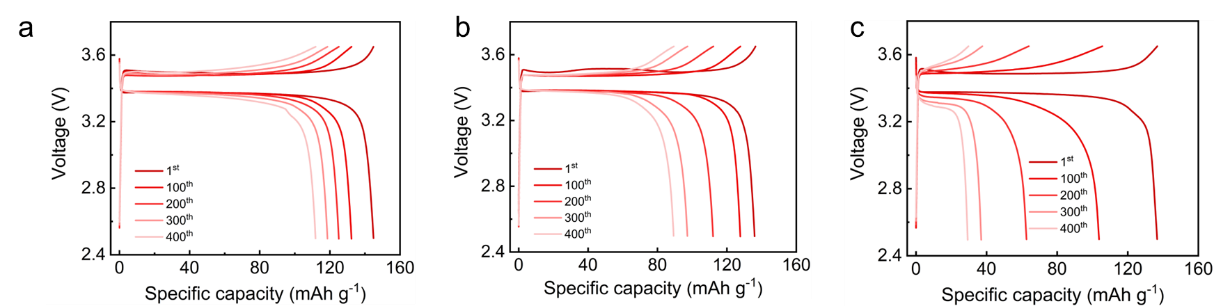


**Fig. S16** Charge-discharge voltage profiles of LFP||Li cell with (**a**) HNT**^+^**-NCCPE, (**b**) HNT-CPE and (**c**) PVDF electrolyte at 0.5C


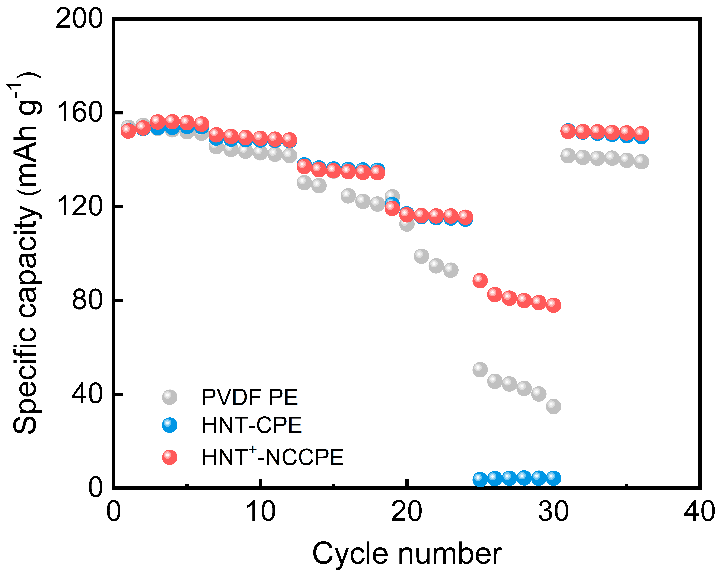


**Fig. S17** Rate capability of LFP||Li cells with PVDF electrolyte, HNT-CPE and HNT**^+^**-NCCPE


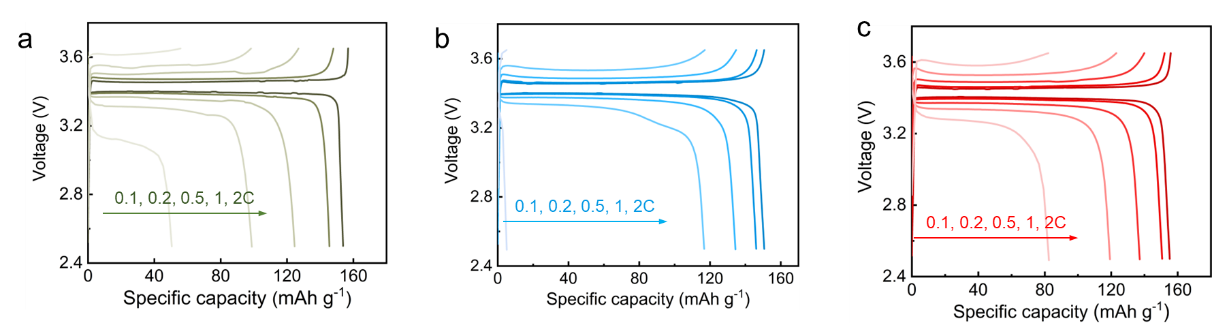


**Fig. S18** Charge-discharge voltage profiles of LFP||Li cell with (**a**) PVDF electrolyte, (**b**) HNT-CPE and (**c**) HNT^+^-NCCPE


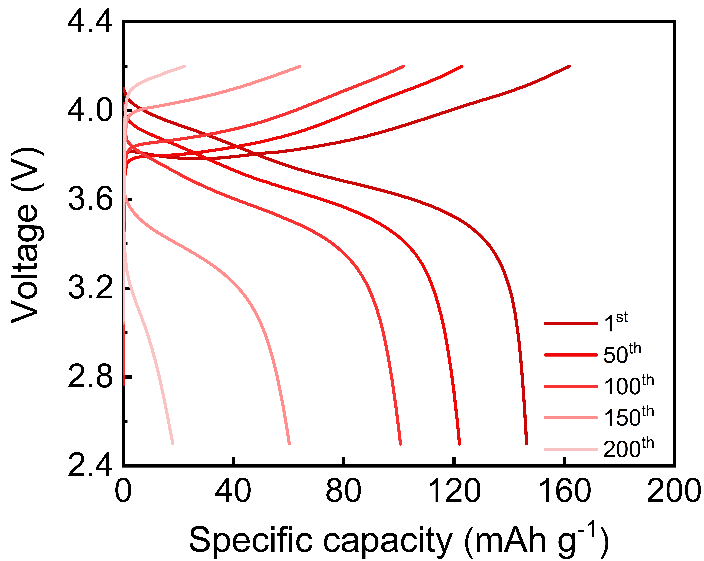


**Fig. S19** Charge-discharge voltage profiles of NCM811||Li cell with PVDF electrolyte at 0.5 C


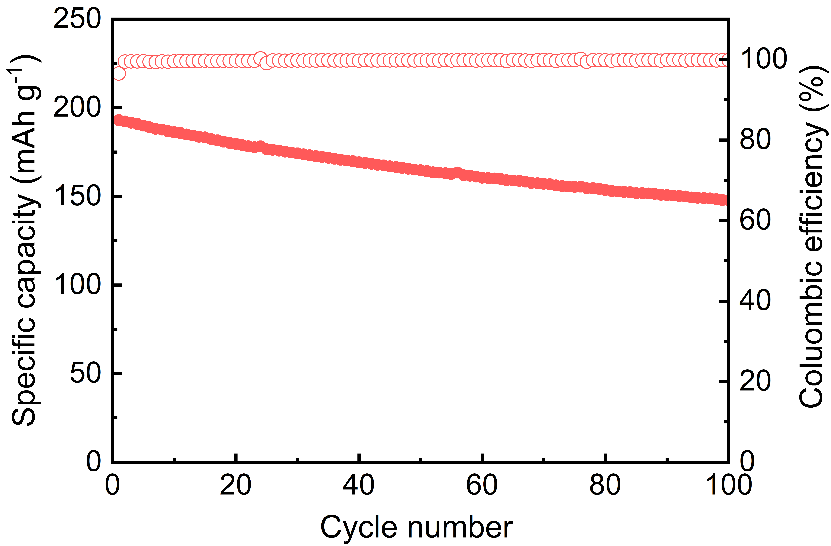


**Fig. S20** Cycling stability of the NCM811|HNT**^+^**-NCCPE|Li cell at 0.5C in the voltage range of 2.8-4.4 V


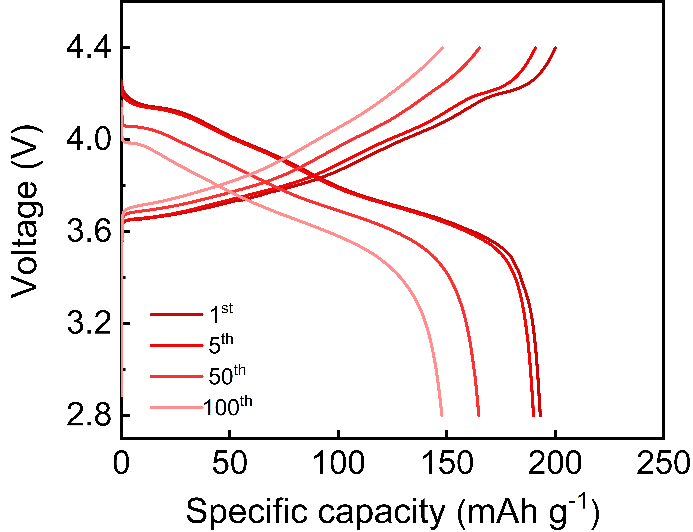


**Fig. S21** Charge-discharge voltage profiles of NCM811|HNT^+^-NCCPE|Li cell at 2.8-4.4 V


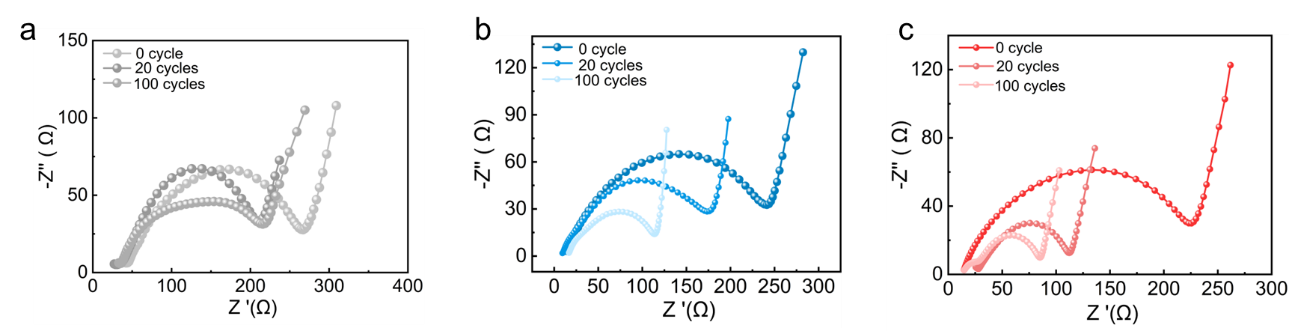


**Fig. S22** Nyquist plots of the NCM811||Li half-cells after cycling with (**a**) PVDF electrolyte, (**b**) HNT-CPE, (**c**) HNT^+^-NCCPE

**Table S1** The p-values and confidence intervals of mechanical strength of various electrolytes

| Sample | H_0_-Strength  (MPa) | p-value | confidence intervals |
| --- | --- | --- | --- |
| HNT-PVDF | 34.70 | 0.36 | [34.57, 35.01] |
| HNT-CPE | 19.13 | 0.57 | [19.04, 19.18] |
| HNT--PVDF | 45.87 | 0.88 | [44.98, 46.89] |
| HNT--NCCPE | 48.39 | 0.97 | [47.67, 49.09] |
| HNT+-PVDF | 45.09 | 0.18 | [45.02, 45.39] |
| HNT+-NCCPE | 32.46 | 0.30 | [31.84, 32.68] |

**Table S2** Comparison of Li^+^ transference number ($t_{{Li}^{+}}$) and mechanical strength of PVDF-based composite solid electrolytes

| PVDF-based electrolyte | $t_{{Li}^{+}}$ | Strength (MPa) | References |
| --- | --- | --- | --- |
| h-PAN@MOF  /PVDF/LiFSI | 0.48 | 20.84 | *Energy Environ. Sci., 2024,17, 8274-8283* [S1] |
| PVDF/PVA@STO/LiFSI | 0.43 | 8.02 | *Energy Environ. Sci., 2025,18, 3730-3739* [S2] |
| PDOL@ZnO/PVDF-HFP | 0.59 | 15.8 | *Adv. Energy Mater. 2025, 2501379* [S3] |
| BTO-MS HNFs/PVDF | 0.61 | 1.90 | *Adv. Energy Mater. 2025, 15, 2405220* [S4] |
| F-PVDF | 0.69 | 6.3 | *Adv. Mater. 2025, 2504419* [S5] |
| PI-PVDF-VS | 0.52 | 8.2 | *Angew. Chem. Int. Ed. 2025, e202423227* [S6] |
| [EMIM][TFSI]@LZSP-PVDF | 0.81 | ≈11 | *J. Am. Chem. Soc. 2024, 146, 10, 6591-6603* [S7] |
| BTO–LLTO-PVDF | 0.57 | 2.11 | *Nat. Nanotechnol.2023* 18, 602–610 [S8] |
| **HNT^+^-NCCPE** | **0.86** | **32.46** | ***This work*** |

**Table S3** Comparison of Li||Li symmetric battery performance

| Electrolyte | Performance | References |
| --- | --- | --- |
| PVDF/LiTFSI/LLZTO@PDA | 0.1 mA cm^-2^-0.1 mAh cm^-2^ for 1000 h | Adv. Energy Mater. 2023, 13, 220437737 [S9] |
| PVDF/LiTFSI-LiDFOB-LiBF4 | 0.25 mA cm^-2^-0.25 mAh cm^-2^ for 270 h | *J. Am. Chem. Soc.* 2023,145, 47, 25632-2564233 [S10] |
| PVDF/LiTFSI/DMIm | 0.1 mA cm^-2^-0.05 mAh cm^-2^ for 800 h | *Angew. Chem. Int. Ed.*  2022, 61, e20220507536 [S11] |
| P(VDF-TrFE-CTFE)  /(Pyr13-TFSI) | 0.1 mA cm^-2^-0.05 mAh cm^-2^ for 1500 h | *Angew. Chem. Int. Ed.*  2023, 62, e20230024338 [S12] |
| PVDF-HFP-LiTFSI-Fluorinated graphene | 0.2 mA cm^-2^-0.1 mAh cm^-2^ for 500 h | Adv. Energy Mater. 2022, 2200967 [S13] |
| PVDF-HFP-LiTSI-(La_0.7_Sr_0.3_)_0.97_TiO_3_ | 0.2 mA cm^-2^-0.1 mAh cm^-2^ for 480 h | Adv. Mater. 2025, 37, 2419782 [S14] |
| P(VDF-TrFE-CTFE)-LiTFSI | 0.05 mA cm^-2^ 0.05 mA cm^-2^ for 1200 h | Energy Environ. Sci., 2021, 14, 6021–6029 [S15] |
| P(VDF-TrFE-CTFE)-LiFSI | 0.1 mA cm^-2^ 0.1 mA cm^-2^  for 1200 h | Adv. Energy Mater. 2023, 2203888 [S16] |
| PVDF-HFP/LiTFSI | 0.3 mA cm^-2^-0.15 mAh cm^-2^ for 300 h | Angew Chem Int Ed 2021; 60: 12931-1294038 [S17] |
| PEO-LiTFSI-BMI-Br | 0.3 mA cm^-2^ for 500 h | Adv. Energy Mater. 2023, 13, 2301674 [S18] |
| β-zeolite-DBDPE-  LiTFSI-PVDF | 0.1 mA cm^-2^-5500 h | *Small 2025, 21, 2406200* [S19] |
| PVDF-LiTFSI-LiDFOB-BTO | 0.2 mA cm^-2^-0.1 mAh cm^-2^ for 5000 h | *ACS Nano* 2025, 19, 3, 3197–3209 [S20] |
| **HNT^+^-NCCPE** | **0.2 mA cm^-2^-0.2 mAh cm^-2^ for 700 h** | **This work** |
| **HNT^+^-NCCPE** | **0.25 mA cm^-2^-0.25 mAh cm^-2^ for 400 h** | **This work** |

**Table S4** Comparison of solid-state battery performance

| Electrolyte | Areal Capacity  (mAh cm^-2^) | | Current density (mA cm^-2^) | Cathode  material | Cycle number | References |
| --- | --- | --- | --- | --- | --- | --- |
| PVDF/LiClO_4_/  LLZTO | | 0.342 | 0.1 | LCO | 120 | *J Am Chem Soc 2017;139: 13779-13785* [S21] |
| PVDF/LiFSI | | 0.504 | 0.15 | LCO | 200 | *Adv Mater 2019; 31: 1806082* [S22] |
| PVDF-HFP/LiTFSI | | 0.378 | 0.075 | NCM532 | 200 | *Angew Chem Int Ed 2021; 60: 12931-1294038* [S17] |
| PVDF-HFP/LiTFSI/SiO_2_ | | 0.935 | 0.187 | LFP | 400 | *Adv Mater 2022; 34: e220557546* [S23] |
| PVDF-HFP/LiTFSI/Fluorinated graphene | | 0.342 | 0.342 | NCM622 | 300 | *Adv Energy Mater 2022; 12: 2200967* [S13] |
| VEC/MASTFSILi/SN | | 0.255 | 0.255 | LFP | 400 | *Adv Mater 2022; 34:*  *e2202143* [S24] |
| IL/VEC/OFHDODA/LiTFSI | | 0.187 | 0.099 | NCM532 | 200 | *Nat Commun 2023; 14: 2301* [S25] |
| PVDF-HFP-LiTSI-(La_0.7_Sr_0.3_)_0.97_TiO_3_ | | 0.45 | 0.53 | LFP | 130 | *Adv. Mater. 2025, 37, 2419782* [S14] |
| COF/LiClO_4_ | | 0.17 | 0.17 | LFP | 750 | *Nat Commun* 2022; 13: 2031 [S26] |
| **HNT^+^-NCCPE** | | **0.45** | **0.26** | **LFP** | **400** | **This work** |

**Table S5** Comparison of electrochemical performance of various electrolytes

| Sample | CE | CCD (mA cm^-2^) | Rct (after 100 cycles) | Capacity retention |
| --- | --- | --- | --- | --- |
| PVDF PE | 80.75 ± 1.95% | 0.1 | 219 | 20.97% |
| HNT-CPE | 84.05 ± 1.40% | 0.5 | 135 | 65.03% |
| HNT^+^-NCCPE | 91.93±0.68% | 1 | 84 | 78.62% |

**Supplementary References**

1. Y. Ma, Y. Qiu, K. Yang, S. Lv, Y. Li et al., Competitive Li-ion coordination for constructing a three-dimensional transport network to achieve ultra-high ionic conductivity of a composite solid-state electrolyte. Energy Environ. Sci. **17**(21), 8274–8283 (2024). <https://doi.org/10.1039/D4EE03134B>
2. Y. Ma, L. Chen, Y. Li, B. Li, X. An et al., Mesoscale polymer regulation for fast-charging solid-state lithium metal batteries. Energy Environ. Sci. **18**(8), 3730–3739 (2025). <https://doi.org/10.1039/d5ee00203f>
3. H. Gao, Y. Zhou, K. Wang, B. Li, S. Wang et al., An *in situ* polymerized solid-state electrolyte for uniform lithium deposition *via* the piezoelectric effects. Adv. Energy Mater. **15**(28), 2570123 (2025). <https://doi.org/10.1002/aenm.202570123>
4. J. Shan, R. Gu, J. Xu, S. Gong, S. Guo et al., Heterojunction ferroelectric materials enhance ion transport and fast charging of polymer solid electrolytes for lithium metal batteries. Adv. Energy Mater. **15**(18), 2405220 (2025). <https://doi.org/10.1002/aenm.202405220>
5. X. Han, J. Lu, Q. Zou, H. Wang, M. Chen et al., Salt-segregated solid polymer electrolytes for high-rate solid-state lithium batteries. Adv. Mater. **37**(24), e2504419 (2025). <https://doi.org/10.1002/adma.202504419>
6. Z. Yang, B. Yang, S. Wang, J. Qian, Z. Hou et al., Multivariate distribution structured anisotropic inorganic polymer composite electrolyte for long-cycle and high-energy all-solid-state lithium metal batteries. Angew. Chem. Int. Ed. **64**(30), e202423227 (2025). <https://doi.org/10.1002/anie.202423227>
7. L. Zhu, J. Chen, Y. Wang, W. Feng, Y. Zhu et al., Tunneling interpenetrative lithium ion conduction channels in polymer-in-ceramic composite solid electrolytes. J. Am. Chem. Soc. **146**(10), 6591–6603 (2024). <https://doi.org/10.1021/jacs.3c11988>
8. P. Shi, J. Ma, M. Liu, S. Guo, Y. Huang et al., A dielectric electrolyte composite with high lithium-ion conductivity for high-voltage solid-state lithium metal batteries. Nat. Nanotechnol. **18**(6), 602–610 (2023). <https://doi.org/10.1038/s41565-023-01341-2>
9. Y. Xu, K. Wang, X. Zhang, Y. Ma, Q. Peng et al., Improved Li-ion conduction and (electro)chemical stability at garnet-polymer interface through metal-nitrogen bonding. Adv. Energy Mater. **13**(14), 2204377 (2023). <https://doi.org/10.1002/aenm.202204377>
10. M. Li, H. An, Y. Song, Q. Liu, J. Wang et al., Ion–dipole-interaction-induced encapsulation of free residual solvent for long-cycle solid-state lithium metal batteries. J. Am. Chem. Soc. **145**(47), 25632–25642 (2023). <https://doi.org/10.1021/jacs.3c07482>
11. X. Pei, Y. Li, T. Ou, X. Liang, Y. Yang et al., Li–N interaction induced deep eutectic gel polymer electrolyte for high performance lithium-metal batteries. Angew. Chem. Int. Ed. **61**(31), e202205075 (2022). <https://doi.org/10.1002/anie.202205075>
12. J.-F. Liu, Z.-Y. Wu, F.J. Stadler, Y.-F. Huang, High dielectric poly(vinylidene fluoride)-based polymer enables uniform lithium-ion transport in solid-state ionogel electrolytes. Angew. Chem. Int. Ed. **62**(26), e202300243 (2023). <https://doi.org/10.1002/anie.202300243>
13. P. Zhai, Z. Yang, Y. Wei, X. Guo, Y. Gong, Two-dimensional fluorinated graphene reinforced solid polymer electrolytes for high-performance solid-state lithium batteries. Adv. Energy Mater. **12**(42), 2200967 (2022). <https://doi.org/10.1002/aenm.202200967>
14. Y.-L. Liao, X.-L. Wang, H. Yuan, Y.-J. Li, C.-M. Xu et al., Ultrafast Li-rich transport in composite solid-state electrolytes. Adv. Mater. **37**(10), e2419782 (2025). <https://doi.org/10.1002/adma.202419782>
15. Y.-F. Huang, T. Gu, G. Rui, P. Shi, W. Fu et al., A relaxor ferroelectric polymer with an ultrahigh dielectric constant largely promotes the dissociation of lithium salts to achieve high ionic conductivity. Energy Environ. Sci. **14**(11), 6021–6029 (2021). <https://doi.org/10.1039/D1EE02663A>
16. Y.-F. Huang, J.-P. Zeng, S.-F. Li, C. Dai, J.-F. Liu et al., Conformational regulation of dielectric poly(vinylidene fluoride)-based solid-state electrolytes for efficient lithium salt dissociation and lithium-ion transportation. Adv. Energy Mater. **13**(15), 2203888 (2023). <https://doi.org/10.1002/aenm.202203888>
17. W. Liu, C. Yi, L. Li, S. Liu, Q. Gui et al., Designing polymer-in-salt electrolyte and fully infiltrated 3D electrode for integrated solid-state lithium batteries. Angew. Chem. Int. Ed. **60**(23), 12931–12940 (2021). <https://doi.org/10.1002/anie.202101537>
18. E. Kim, H. Jamal, I. Jeon, F. Khan, S.-E. Chun et al., Functionality of 1-butyl-2, 3-dimethylimidazolium bromide (BMI-Br) as a solid plasticizer in PEO-based polymer electrolyte for highly reliable lithium metal batteries (adv. energy mater. 47/2023). Adv. Energy Mater. **13**(47), 2370192 (2023). <https://doi.org/10.1002/aenm.202370192>
19. H. Lim, M.S. Chae, H. Jamal, F. Khan, I. Jeon et al., Triple-layered noncombustible PEO-based solid electrolyte for highly safe lithium-metal batteries. Small **21**(14), e2406200 (2025). <https://doi.org/10.1002/smll.202406200>
20. Y. Zhang, H. Liu, F. Liu, S. Zhang, M. Zhou et al., Dual-anion-rich polymer electrolytes for high-voltage solid-state lithium metal batteries. ACS Nano **19**(3), 3197–3209 (2025). <https://doi.org/10.1021/acsnano.4c09953>
21. X. Zhang, T. Liu, S. Zhang, X. Huang, B. Xu et al., Synergistic coupling between Li_6.75_La_3_Zr_1.75_Ta_0.25_O_12_ and poly(vinylidene fluoride) induces high ionic conductivity, mechanical strength, and thermal stability of solid composite electrolytes. J. Am. Chem. Soc. **139**(39), 13779–13785 (2017). <https://doi.org/10.1021/jacs.7b06364>
22. X. Zhang, S. Wang, C. Xue, C. Xin, Y. Lin et al., Self-suppression of lithium dendrite in all-solid-state lithium metal batteries with poly(vinylidene difluoride)-based solid electrolytes. Adv. Mater. **31**(11), 1806082 (2019). <https://doi.org/10.1002/adma.201806082>
23. T. Zhang, J. Li, X. Li, R. Wang, C. Wang et al., A silica-reinforced composite electrolyte with greatly enhanced interfacial lithium-ion transfer kinetics for high-performance lithium metal batteries. Adv. Mater. **34**(41), 2205575 (2022). <https://doi.org/10.1002/adma.202205575>
24. K. Wen, C. Xin, S. Guan, X. Wu, S. He et al., Ion–dipole interaction regulation enables high-performance single-ion polymer conductors for solid-state batteries. Adv. Mater. **34**(32), 2202143 (2022). <https://doi.org/10.1002/adma.202202143>
25. L. Tang, B. Chen, Z. Zhang, C. Ma, J. Chen et al., Polyfluorinated crosslinker-based solid polymer electrolytes for long-cycling 4.5 V lithium metal batteries. Nat. Commun. **14**(1), 2301 (2023). <https://doi.org/10.1038/s41467-023-37997-6>
26. J. Li, J. Qi, F. Jin, F. Zhang, L. Zheng et al., Room temperature all-solid-state lithium batteries based on a soluble organic cage ionic conductor. Nat. Commun. **13**(1), 2031 (2022). <https://doi.org/10.1038/s41467-022-29743-1>
